# Identification of a Functional *CYP2C8* Variant Allele that Alters Splicing, Reduces Protein Expression, and Increases Drug Exposure

**DOI:** 10.1002/cpt.70376

**Published:** 2026-07-01

**Authors:** Anssi J. H. Mykkänen, Päivi Hirvensalo, Kathrin Klein, Dorota Kaminska, Markus Grube, Tuija Tapaninen, Johanna I. Kiiski, Mikko Neuvonen, Ville T. Männistö, Jussi Pihlajamäki, Mladen Tzvetkov, Matthias Schwab, Aleksi Tornio, Janne T. Backman, Mikko Niemi

**Affiliations:** ^1^ Department of Clinical Pharmacology University of Helsinki Helsinki Finland; ^2^ Department of Clinical Pharmacology, HUS Diagnostic Center Helsinki University Hospital Helsinki Finland; ^3^ Individualized Drug Therapy Research Program University of Helsinki Helsinki Finland; ^4^ Integrative Physiology and Pharmacology, Institute of Biomedicine University of Turku Turku Finland; ^5^ Dr. Margarete Fischer‐Bosch Institute of Clinical Pharmacology Stuttgart Germany; ^6^ University of Tuebingen Tuebingen Germany; ^7^ Institute of Public Health and Clinical Nutrition University of Eastern Finland Kuopio Finland; ^8^ Department of Medicine, Division of Digestive Diseases UCLA Los Angeles California USA; ^9^ Department of General Pharmacology University Medicine Greifswald Greifswald Germany; ^10^ Institute of Clinical Medicine University of Eastern Finland and Kuopio University Hospital Kuopio Finland; ^11^ Department of Medicine, Endocrinology and Clinical Nutrition Kuopio University Hospital Kuopio Finland; ^12^ Departments of Clinical Pharmacology, and of Biochemistry and Pharmacy University Tuebingen Tuebingen Germany; ^13^ Unit of Clinical Pharmacology Turku University Hospital Turku Finland

## Abstract

This study investigated genetic determinants of the pharmacokinetics of the CYP2C8 index drugs repaglinide and gemfibrozil, and their interaction in healthy participants. Sequencing data from a study with montelukast revealed a novel functional *CYP2C8* allele (rs2071426, CYP2C8*19), predicted to create an intronic splice donor site. In human liver samples, CYP2C8*19 associated with transcript‐specific changes in *CYP2C8* mRNA expression, reduced CYP2C8 protein expression, and decreased enzyme activity. Consistently, participants with the CYP2C8*19/*19 genotype had 45% greater area under the plasma repaglinide concentration–time curve from time zero to infinity (AUC_0‐∞_) than participants with CYP2C8*1/*1 (*P* = 1.6 × 10^−4^). Participants with CYP2C8*1/*3 had 26% smaller AUC_0‐∞_ (*P* = 0.0033) and those with CYP2C8*1/*4 had 51% greater AUC_0‐∞_ (*P* = 8.2 × 10^−4^). The fold increase in repaglinide AUC_0‐∞_ caused by gemfibrozil was 36% (*P* = 1.3 × 10^−4^) smaller in CYP2C8*19/*19 participants than in CYP2C8*1/*1 participants. In a genome‐wide association study (GWAS), *SLCO1B1* c.521 T>C (rs4149056) associated with increased repaglinide AUC_0‐∞_ (*P* = 4.5 × 10^−15^; *n* = 172) and *SLCO1A2* variants associated with decreased AUC_0‐∞_ (*P* < 10^−8^). In a GWAS of repaglinide after gemfibrozil pretreatment, *SLCO1C1* variants associated with decreased AUC_0‐∞_ (*P* < 1.6 × 10^−8^; *n* = 66). Participants with the poor function *SLCO1B1* genotype showed a 32% smaller fold increase in repaglinide AUC_0‐∞_ following gemfibrozil than participants with the normal function *SLCO1B1* genotype (*P* = 0.0045). This study characterizes CYP2C8*19 as a novel decreased function allele and shows that *CYP2C8* and *SLCO1B1* genotypes affect the gemfibrozil–repaglinide interaction.

**Study Highlights**

**WHAT IS THE CURRENT KNOWLEDGE ON THE TOPIC?**

Repaglinide is an index CYP2C8 substrate. Considerable variability exists in repaglinide pharmacokinetics, and genetic variation explains part of the variability. Gemfibrozil is an index CYP2C8 inhibitor, which increases the AUC_0‐∞_ of repaglinide ~ 7–8‐fold.

**WHAT QUESTION DID THIS STUDY ADDRESS?**

What are the most important genetic variants influencing the pharmacokinetics of repaglinide and gemfibrozil and their interaction?

**WHAT DOES THIS STUDY ADD TO OUR KNOWLEDGE?**

This study characterized a novel *CYP2C8* allele, CYP2C8*19, which results in an alternative *CYP2C8* transcript, reduces the protein expression of CYP2C8, and increases the exposure to CYP2C8 substrates. In addition, genome‐wide significant associations were observed between *SLCO1B1* and *SLCO1A2* variants and repaglinide pharmacokinetics without concomitant medications, and between *SLCO1C1* variants and repaglinide pharmacokinetics after gemfibrozil pretreatment.

**HOW MIGHT THIS CHANGE CLINICAL PHARMACOLOGY OR TRANSLATIONAL SCIENCE?**

This study describes a novel decreased function *CYP2C8* allele and elucidates the roles of *SLCO1B1* and *CYP2C8* in interindividual variability of the pharmacokinetics of repaglinide and the gemfibrozil–repaglinide interaction. Pharmacogenetic variation should be considered in the interpretation of drug–drug interaction studies.

Repaglinide and gemfibrozil are recommended as an index substrate and inhibitor, respectively, for investigating cytochrome P450 (CYP) 2C8‐mediated drug–drug interactions in humans.^1^ Repaglinide undergoes extensive metabolism primarily by CYP2C8 to inactive metabolites, with a minor contribution by CYP3A4.^2^, ^3^, ^4^, ^5^, ^6^, ^7^ Repaglinide is also a substrate for organic anion‐transporting polypeptide (OATP) 1B1, encoded by *SLCO1B1*, an influx transporter which transports repaglinide from blood into the hepatocytes.^8^, ^9^ Gemfibrozil is metabolized by the UDP‐glucuronosyltransferase 2B7 enzyme into gemfibrozil 1‐O‐β‐glucuronide, which is a strong metabolism‐dependent inhibitor of CYP2C8.^3^, ^10^, ^11^, ^12^, ^13^, ^14^, ^15^, ^16^, ^17^, ^18^, ^19^, ^20^ Moreover, gemfibrozil is a weak OATP1B1 inhibitor.^3^, ^21^ Concomitant gemfibrozil use causes a 7–8‐fold increase in the AUC_0‐∞_ of repaglinide.^4^, ^10^, ^11^, ^12^, ^22^, ^23^, ^24^

Genetic variation influences the pharmacokinetics of repaglinide. It is well established that the *SLCO1B1* c.521 T>C (p.Val174Ala, rs4149056) no function variant of OATP1B1 increases exposure to repaglinide.^8^, ^25^, ^26^ However, the role of genetic variability in CYP2C8 activity is unclear. Some studies have reported that the CYP2C8*3 allele, defined by c.416G>A (p.Arg139Lys, rs11572080) and c.1196A>G (p.Lys399Arg, rs10509681), is associated with reduced plasma concentrations of repaglinide.^3^, ^8^, ^27^ In contrast, the impact of genetic variation on gemfibrozil pharmacokinetics has not been investigated.

Genetic variation may confound the assessment of drug–drug interactions. The aim of this study was to identify genetic variants affecting the pharmacokinetics of the CYP2C8 index drugs repaglinide and gemfibrozil. A genome‐wide association study (GWAS) was used as a hypothesis‐free method to identify variants affecting the pharmacokinetics of repaglinide and gemfibrozil. Because pharmacogenetic variation of CYP2C8 is not well established, genetic variation of CYP2C8 was further investigated with a candidate gene analysis. Data from a previous pharmacogenetic study with another CYP2C8 substrate, montelukast, and high throughput sequencing of the *CYP2C* locus was used to identify potential novel functional *CYP2C8* variants.

## METHODS

### Participants

A total of 252 unrelated healthy white Finnish individuals were included from previous pharmacokinetic studies, with 172 participants from studies with repaglinide and 158 from studies with gemfibrozil (**Table** **S1**, **Table** **S2**). Of the participants, 66 were from gemfibrozil–repaglinide interaction studies. The participants' mean ± standard deviation age was 23.1 ± 2.9 years and weight 72.0 ± 11.4 kg. The subjects were ascertained to be healthy by medical history, clinical examination, and laboratory tests before entering the studies. None was a tobacco smoker or used any continuous medication.

### Genotyping

During the pharmacokinetic studies, a whole blood sample was collected from each participant into an ethylenediaminetetraacetic acid (EDTA)‐containing tube for DNA extraction. For this study, genomic DNA was extracted using the Maxwell 16 LEV Blood DNA Kit on a Maxwell 16 Research automated nucleic acid extraction system (Promega, Madison, WI) and genome‐wide genotyping was carried out at the Institute for Molecular Medicine Finland (Helsinki, Finland) using Illumina HumanCoreExome‐24v1‐1_A BeadChip (Illumina, San Diego, CA). Hardy–Weinberg equilibrium *P* > 10^−5^ and proportion missing ≤ 0.03 were employed as quality thresholds for including genotype data in statistical analysis. A total of 260,995 single‐nucleotide variations (SNV) with MAF ≥ 0.05 were included in the genome‐wide analyses. First‐ and second‐degree relatives were identified with identity by descent analysis and excluded from further analyses. A principal component analysis was carried out to identify genetic outliers, who were excluded from analyses. To supplement missing data for the candidate gene analysis, the participants were genotyped for selected variants with TaqMan genotyping assays on a QuantStudio 12 K Flex Real‐Time PCR system (Thermo Fisher Scientific, Waltham, MA). GWAS top hit and known or suspected functional variants of *SLCO1B1* and *CYP2C8* with a MAF ≥ 0.01 were included in the candidate gene analyses (**Table** **S3**). Haplotypes were computed with PHASE v2.1.1.^28^, ^29^ For determination of functional *SLCO1B1* genotype groups, *SLCO1B1* haplotypes were defined based on the c.388A>G (rs2306283), c.463C>A (rs11045819), c.521 T>C (rs4149056), and c.1929A>C (rs34671512) SNVs according to Pharmacogene Variation Consortium.^30^ *SLCO1B1* genotype function was assigned as described previously.^31^

### Pharmacokinetics

Repaglinide and gemfibrozil plasma concentrations were obtained from the previous pharmacokinetic studies (**Table** **S1**, **Table** **S2**). Studies on repaglinide were either drug–drug interaction studies where repaglinide was the victim drug or pharmacogenetic studies. The participants had ingested either a 0.25‐mg (*n* = 151) or a 0.5‐mg (*n* = 21) dose of repaglinide (NovoNorm, Novo Nordisk, Bagsværd, Denmark) with 150 mL water in the morning between 8.00 and 9.30 a.m. after an overnight fast. Timed blood samples were drawn before and up to 7–12 h after repaglinide ingestion for the determination of repaglinide plasma concentrations. If a participant had participated in multiple studies, studies with the 0.25 mg dose of repaglinide, gemfibrozil–repaglinide interaction studies, and the most recent studies were prioritized in data selection.

Studies on gemfibrozil were drug–drug interaction studies where gemfibrozil was the perpetrator drug (**Table** **S1**, **Table** **S2**). In most of the studies, gemfibrozil (Lopid 600 mg tablet; Gödecke, Freiburg, Germany) was administered at a dose of 600 mg b.i.d. for 2 days prior to the study day (i.e., five doses in total). In the gemfibrozil–repaglinide interaction studies, gemfibrozil was administered 1 h before repaglinide, except for one study in which it was administered at the same time with repaglinide.^11^ In two studies, the gemfibrozil dose was 900 mg^11^, ^24^ and in two studies gemfibrozil was administered as a single dose.^11^, ^12^ Gemfibrozil 1‐O‐β glucuronide concentrations were available for 98 of the 158 participants. Studies with a gemfibrozil dose of 600 mg, gemfibrozil–repaglinide interaction studies, and studies with a total of five doses of gemfibrozil were prioritized in data selection.

The plasma concentrations of repaglinide and gemfibrozil had been quantified by use of an API 3000 or a 5500 Qtrap liquid chromatography–tandem mass spectrometry system (AB Sciex, Toronto, ON, Canada), as described previously (**Table** **S2**). The lower limit of quantification was 0.01–0.1 ng/mL for repaglinide and 2.5–250 ng/mL for gemfibrozil and gemfibrozil 1‐O‐β‐glucuronide (**Table** **S2**). The day‐to‐day coefficient of variation (CV) was below 15% at relevant concentrations for all analytes.

Pharmacokinetic variables were recalculated with standard noncompartmental methods (Phoenix® WinNonlin®, version 6.4; Certara, Princeton, NJ). Repaglinide area under the plasma concentration–time curve from time zero to infinity (AUC_0‐∞_) was calculated without concomitant medication and after gemfibrozil pretreatment. Repaglinide AUC_0‐∞_ values for participants from studies with the 0.5 mg dose of repaglinide were linearly adjusted to the 0.25 mg dose. For gemfibrozil and gemfibrozil 1‐O‐β glucuronide, the between dose AUC (AUC_0‐12h_) was calculated for participants from studies with multiple doses of gemfibrozil, and the AUC_0‐∞_ was calculated for participants from single‐dose studies. For studies with a 900 mg dose of gemfibrozil, gemfibrozil and gemfibrozil 1‐O‐β glucuronide AUC values were adjusted to the 600 mg dose as described previously.^11^ AUC values were calculated using the linear up—logarithmic down method, with extrapolation to infinity by dividing the last predicted concentration by the elimination rate constant.

### Statistical analyses for repaglinide and gemfibrozil

The data were analyzed using the statistical programs JMP Pro 17.2 and 18.0 (SAS Institute, Cary, NC) and IBM SPSS Statistics for Windows 29.0 and 30.0 (Armonk, NY). Before statistical analysis, body weight and pharmacokinetic variables were log‐transformed. Covariates were identified using a forward stepwise linear regression analysis. The tested variables were sex and body weight for all pharmacokinetic variables, unadjusted AUC_0‐12h_ of gemfibrozil and gemfibrozil 1‐O‐β‐glucuronide for repaglinide after gemfibrozil pretreatment, and number of gemfibrozil doses for gemfibrozil and gemfibrozil 1‐O‐β‐glucuronide. *P*‐value thresholds of 0.05 and 0.10 were employed for entry into and removal from the model.

Associations of genetic variants with the pharmacokinetic variables of repaglinide and gemfibrozil were investigated in a GWAS using stepwise linear regression analysis, with covariates set as fixed factors. In case of missing genotype data, cases were excluded pairwise. A *P*‐value of < 5 × 10^−8^ was considered genome‐wide significant.

A general linear model was used to analyze the associations of *SLCO1B1*, *CYP2C8*, and *SLCO1C1* genotype groups with the pharmacokinetic variables of repaglinide without concomitant medication and after gemfibrozil pretreatment. Genotype groups with less than three participants were excluded from the analyses. Pharmacokinetic variables were set as dependent variables with appropriate covariates and genotype groups as fixed factors. Pairwise comparisons were performed with the Fisher's least significant difference method, and a *P*‐value < 0.05 was considered statistically significant.

### OATP1A2 transport

The transport of repaglinide by OATP1A2 was investigated in MDCKII cells stably transfected with human OATP1A2, essentially as described previously.^32^ Repaglinide concentrations were quantified by use of a 4000 Qtrap liquid chromatography–tandem mass spectrometry system (AB Sciex).

### OATP1B1 and OATP1B3 biomarkers

Associations of the *SLCO1C1* rs10841611 SNV with the OATP1B1 biomarker glycochenodeoxycholate 3‐O‐glucuronide (GCDCA‐3G) and the OATP1B3 biomarker glycochenodeoxycholic acid 3‐O‐sulfate (GCDCA‐S) were investigated in 356 healthy participants with previously determined biomarker and genotype data.^33^, ^34^ Statistical comparisons were carried out using a general linear model, with *SLCO1B1* genotype as a fixed factor for GCDCA‐3G.^31^

### Identification of functional *CYP2C8* variants

To discover novel functional variants, genetic variation of *CYP2C8* was investigated in 191 healthy participants from a previous pharmacogenetic study with the CYP2C8 substrate montelukast and high throughput sequencing of the *CYP2C* locus.^35^ Associations of *CYP2C8* variants (MAF > 5%) with montelukast AUC_0‐∞_ were investigated with linear regression analysis as described previously.^36^ The impact of the *CYP2C8* variant that showed the strongest association with montelukast AUC_0‐∞_, rs2071426, was further investigated in a stepwise linear regression analysis with other genes affecting the pharmacokinetics of montelukast, as described previously.^36^

### Human liver samples

The associations of *CYP2C8* rs2071426 with *CYP2C8* messenger RNA (mRNA) expression, and CYP2C8 protein expression and activity were investigated in a human liver cohort collected from 150 patients. In the same cohort, the associations of *SLCO1C1* rs10841611 were investigated with mRNA expression of *SLCO1B1*, *SLCO1B3*, and *SLCO1C1* and protein expression of OATP1B1 and OATP1B3. CYP2C8 genotypes, mRNA, and *in vitro* amodiaquine N‐desethylation activity were determined as described previously.^37^ CYP2C8 protein was quantified by targeted proteomics using mass spectrometry.^38^ OATP1B1 and OATP1B3 proteins were determined with immunoblot analyses.^39^ Statistical comparisons were univariate Wilcoxon or Kruskall–Wallis tests with *P* < 0.05 considered statistically significant. Analyses were performed in R (version 4.5.1).

The associations of *CYP2C8* rs2071426 with the expression levels of different *CYP2C8* mRNA transcripts and *SLCO1C1* rs10841611 with the expression levels of *SLCO1B1*, *SLCO1B3*, and *SLCO1C1* transcripts were investigated in bulk RNA‐sequencing data from 265 human liver samples obtained from patients undergoing laparoscopic gastric bypass operation at the Kuopio University Hospital, as part of the Kuopio Obesity Surgery Study.^35^ Transcript‐level expression was quantified as transcripts per million (TPM) from kallisto pseudoalignment against the GRCh38 RefSeq transcriptome. Using the corresponding NCBI GTF annotation (GCF_000001405.40_GRCh38.p14), we restricted the analysis to protein‐coding RefSeq transcripts annotated with “NM_” identifiers and retained only transcripts with a mean TPM ≥ 10 across all samples. Expression values were transformed as log_2_(TPM) prior to analysis. Participants were genotyped using the Illumina OMNI Exome Express array. Analysis of covariance (ANCOVA) was used to assess the association between genotype and transcripts, with the log_2_‐transformed expression as the dependent variable; genotype was set as the main factor and RNA integrity number, age, sex, and body mass index were set as covariates. Analyses were performed in R (version 4.5.2).

### Ethics statement

Each participant gave a written informed consent before participation. The pharmacokinetic studies were approved by the Ethics Committee of the Hospital District of Helsinki and Uusimaa and the Finnish Medicines Agency Fimea. The use of human liver samples and clinical information was approved by the ethics committees of the University Medical Center Charite Berlin and the University Hospital Tübingen. The Kuopio Obesity Surgery Study was approved by the Ethics Committee of the Northern Savo Hospital District.

## RESULTS

### Discovery of a novel functional *CYP2C8* allele

**Figure 1:**
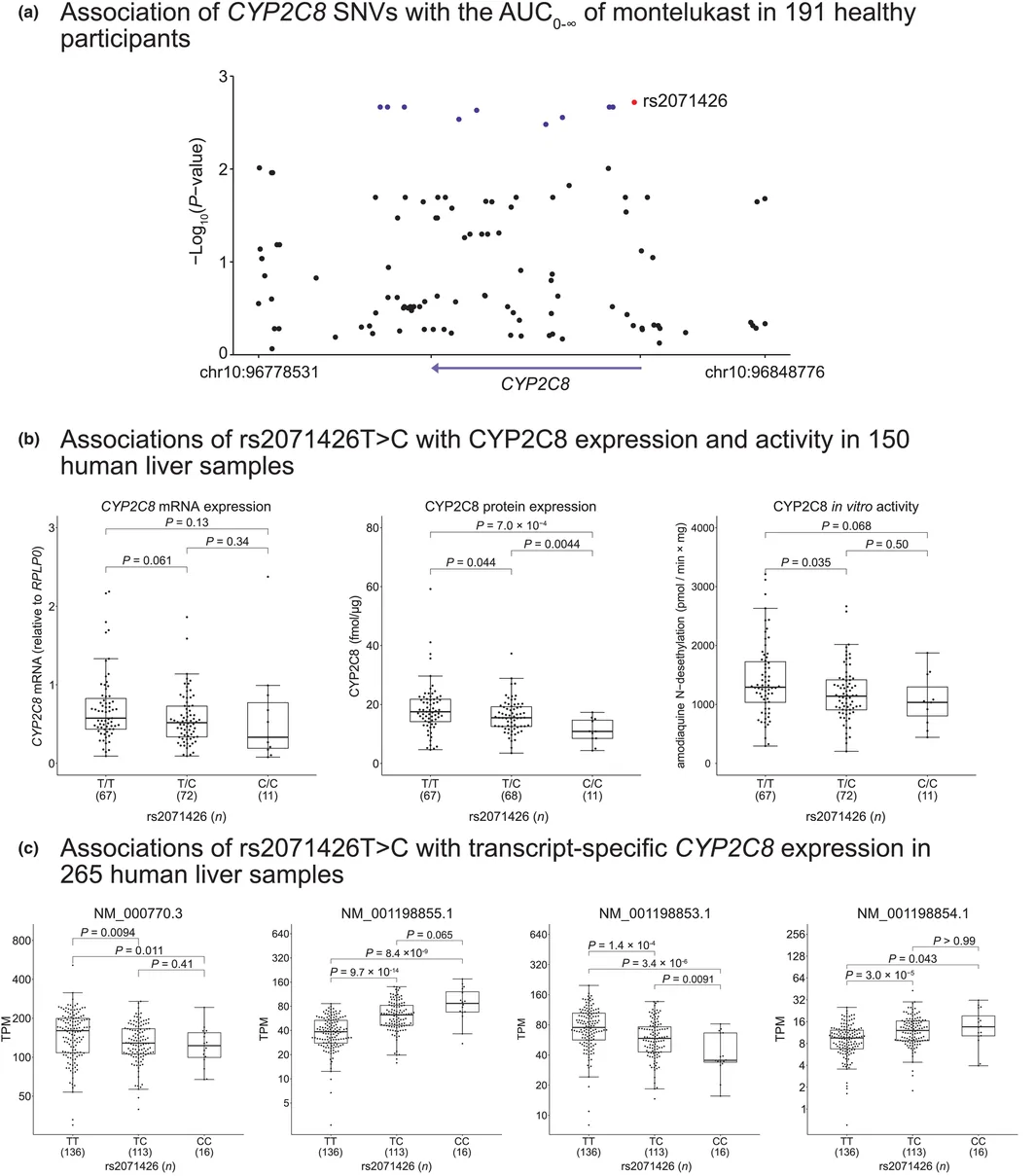
Discovery of *CYP2C8* variants associated with montelukast pharmacokinetics and impact of rs2071426 on CYP2C8 expression and activity in human liver samples. (a) gene‐level association plot of *CYP2C8* variants with montelukast AUC_0‐∞_ in 191 healthy participants; (b) associations of rs2071426 with *CYP2C8* mRNA and protein expression, and *in vitro* activity measured by amodiaquine metabolism; (c) associations of rs2071426 with transcript‐specific *CYP2C8* expression. In (a) dark purple indicates variants strongly linked (*r* ^2^ > 0.96) with rs2071426. In box and whiskers plots (b, c) box lines indicate median with interquartile range, whiskers extend to smallest or largest values within 1.5 × interquartile range from the hinge, and dots represent individual values.

To discover functional *CYP2C8* alleles, targeted sequencing data of the entire *CYP2C8* gene and pharmacokinetic data of the CYP2C8 substrate montelukast were reanalyzed from a previous study (*n* = 191).^36^ An intronic *CYP2C8* SNV (rs2071426) showed the strongest association with montelukast AUC_0‐∞_ (**Figure** **1**). The rs2071426 variant allele was found to reside in a common *CYP2C8* haplotype with no missense variants, herein named as CYP2C8*19 (**Figure** **S1**). When accounting for other genetic variants affecting montelukast, CYP2C8*19 associated with a 13% (90% CI: 7–20%; *P* = 3.7 × 10^−4^) increase in the AUC_0‐∞_ of montelukast per copy of the variant allele (**Table** **S4**).

**Figure 2:**
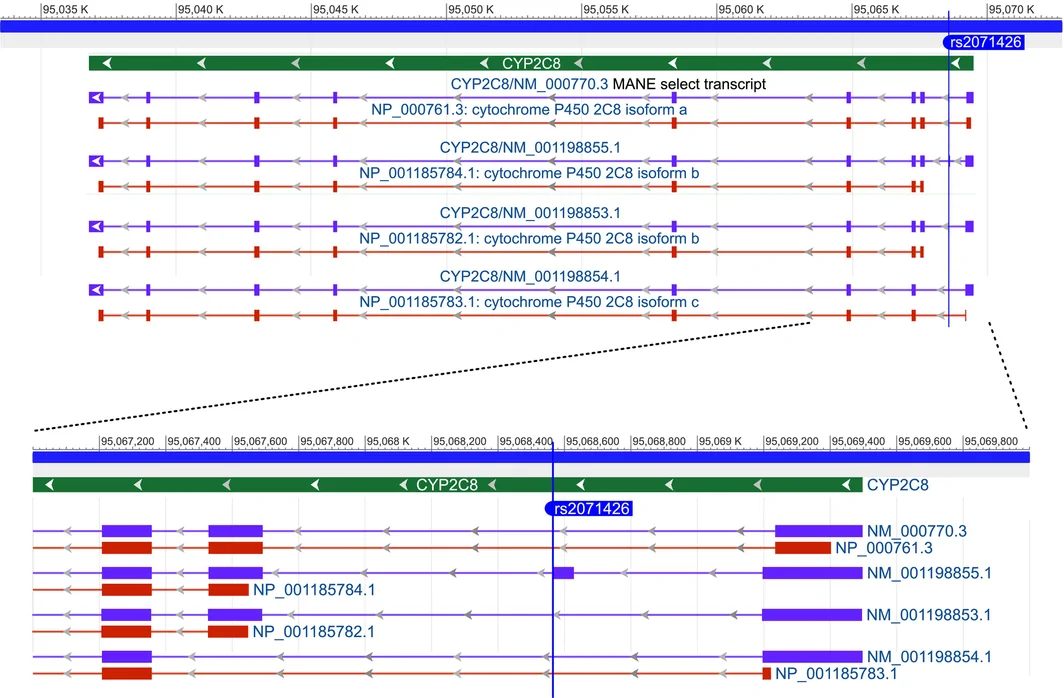
Location of rs2071426 in *CYP2C8* transcripts and protein structure. Data obtained from NCBI Genome Data Viewer (GDV) https://www.ncbi.nlm.nih.gov/gdv/.^40^

*CYP2C8* rs2071426 is located in the first intron of the main protein‐coding transcript of *CYP2C8* (NM_000770.3), and it causes a splice donor site resulting in an alternative transcript with an additional exon (NM_001198855.1) (**Figure** **2**). In the first human liver sample cohort, rs2071426 associated with decreased CYP2C8 protein expression and activity measured with amodiaquine N‐desethylation (**Figure** **1**). In the second cohort, rs2071426 associated with altered expression of different *CYP2C8* transcripts (**Figure** **1**). Expression of NM_001198855.1 containing the additional exon was 63% (90% CI: 47–80%, *P* = 9.7 × 10^−14^) and 119% (90% CI: 78–170%, *P* = 8.4 × 10^−9^) higher in rs2071426 heterozygotes and homozygotes than in noncarriers. In contrast, the expression of the main protein‐coding transcript (NM_000770.3) was 12% (90% CI: 6–19%, *P* = 0.0094) and 24% (90% CI: 11–35%, *P* = 0.011) lower in rs2071426 heterozygotes and homozygotes than in noncarriers. Moreover, rs2071426 associated with decreased expression of NM_001198853.1 and increased expression of NM_001198854.1 (**Figure** **1**).

### GWAS of repaglinide and gemfibrozil pharmacokinetics

**Figure 3:**
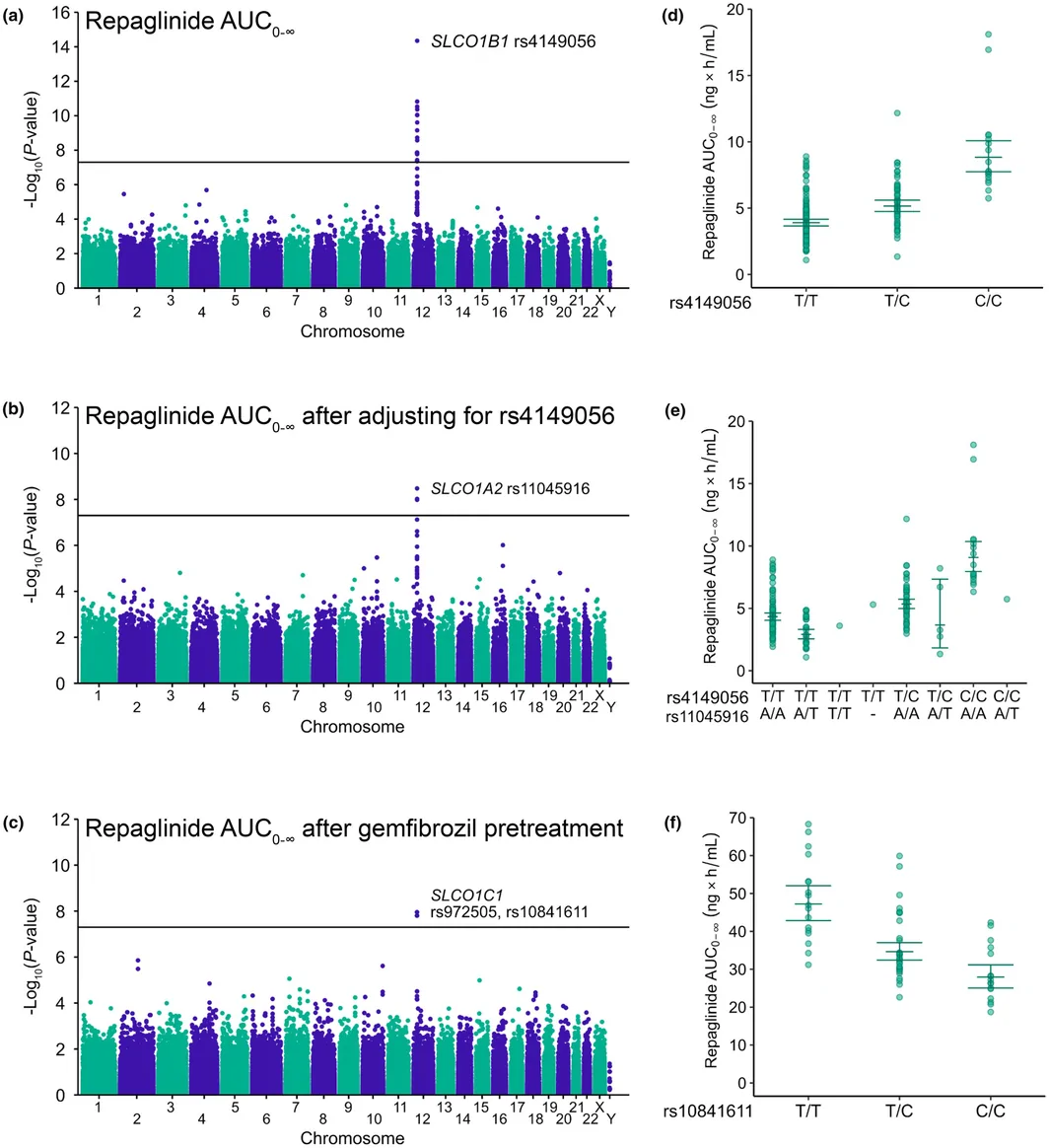
Manhattan plots of (a) repaglinide AUC_0‐∞_ in 172 participants, (b) repaglinide AUC_0‐∞_ after adjusting for rs4149056, and (c) repaglinide AUC_0‐∞_ after gemfibrozil pretreatment in 66 participants. Repaglinide AUC_0‐∞_ values (d) by rs4149056 genotype, (e) by rs4149056 and rs11045916 genotypes, and (f) after gemfibrozil pretreatment by rs10841611 genotype. In (a–c) horizontal lines indicate the genome‐wide significance level of 5 × 10^−8^. In (d–f) horizontal lines indicate geometric means with 90% confidence intervals, and circles indicate individual AUC_0‐∞_ values.

The AUC_0‐∞_ of repaglinide without concomitant medication showed large, 16.5‐fold, between‐subject variability, which was reduced to only 3.7‐fold after gemfibrozil pretreatment (**Table** **S5**). In a GWAS, 21 SNVs in the *SLCO1* region associated genome‐wide significantly with the AUC_0‐∞_ of repaglinide (**Table** **1**, **Figure** **3**). The strongest association was observed between the *SLCO1B1* c.521 T>C (rs4149056, p.Val174Ala) no function variant and an increased AUC_0‐∞_ of repaglinide (*P* = 4.5 × 10^−15^). After accounting for *SLCO1B1* c.521 T>C, six nearly completely linked SNVs in the *SLCO1A2* gene associated genome‐wide significantly with a decreased AUC_0‐∞_ of repaglinide, with a 3′UTR‐variant (rs11045916) showing the strongest association (*P* = 3.3 × 10^−9^). The *SLCO1A2* SNVs were also in a linkage disequilibrium with *SLCO1B1* c.463C>A (p.Pro155Thr, rs11045819), which defines the SLCO1B1*14 increased function allele (**Figure** **S2**). No genome‐wide significant associations were observed after accounting for both *SLCO1B1* c.521 T>C and *SLCO1A2* rs11045916 (**Figure** **S3**).

**Table 1:** Significant associations from genome‐wide association studies

Trait	Analysis	rsID	Nearest gene	Variant type	Nucleotide change	Amino acid change	Effect (90% CI)	*P*	MAF
Repaglinide AUC_0‐∞_	Step 1	rs4149056	*SLCO1B1*	Missense	c.521 T>C	p.Val174Ala	41% (32%, 50%)	4.5 × 10^−15^	0.25
Repaglinide AUC_0‐∞_	Step 1	rs4363657	*SLCO1B1*	Intron	c.1498‐1331 T>C	‐	33% (25%, 42%)	1.5 × 10^−11^	0.33
Repaglinide AUC_0‐∞_	Step 1	rs11045863	*SLCO1B1*	Intron	c.1497 + 2901C>T	‐	−32% (−38%, −26%)	3.0 × 10^−11^	0.13
Repaglinide AUC_0‐∞_	Step 1	rs11045916	*SLCO1A2*	3′UTR	c.*3042 T>A	‐	−37% (−44%, −30%)	4.3 × 10^−11^	0.10
Repaglinide AUC_0‐∞_	Step 1	rs11045891	*SLCO1B1*	3′UTR	c.*449A>C	‐	−33% (−40%, −27%)	9.0 × 10^−11^	0.13
Repaglinide AUC_0‐∞_	Step 1	rs71446763	*SLCO1A2*	3′UTR	c.*4084C>T	‐	−37% (−43%, −29%)	9.2 × 10^−11^	0.10
Repaglinide AUC_0‐∞_	Step 1	rs11045917	*SLCO1A2*	3′UTR	c.*2534C>T	‐	−37% (−43%, −29%)	9.2 × 10^−11^	0.10
Repaglinide AUC_0‐∞_	Step 1	rs11045918	*SLCO1A2*	3′UTR	c.*1770G>T	‐	−37% (−43%, −29%)	9.2 × 10^−11^	0.10
Repaglinide AUC_0‐∞_	Step 1	rs6487215	*SLCO1A2*	Intron	c.1610 + 107C>T	‐	−37% (−43%, −29%)	9.2 × 10^−11^	0.10
Repaglinide AUC_0‐∞_	Step 1	rs10841795	*SLCO1A2*	Missense	c.38 T>C	Ile13Thr	−37% (−43%, −29%)	9.2 × 10^−11^	0.10
Repaglinide AUC_0‐∞_	Step 1	rs11045906	*SLCO1A2*	Intergenic	A>G	‐	−30% (−36%, −24%)	2.5 × 10^−10^	0.16
Repaglinide AUC_0‐∞_	Step 1	rs11045819	*SLCO1B1*	Missense	c.463C>A	p.Pro155Thr	−32% (−39%, −25%)	7.1 × 10^−10^	0.11
Repaglinide AUC_0‐∞_	Step 1	rs10841781	*SLCO1A2*	3′UTR	c.*3087 T>C	‐	−28% (−34%, −22%)	1.9 × 10^−9^	0.16
Repaglinide AUC_0‐∞_	Step 1	rs4149006	*SLCO1A2*	3′UTR	c.*712C>A	‐	−28% (−34%, −22%)	2.7 × 10^−9^	0.16
Repaglinide AUC_0‐∞_	Step 1	rs1304539	*SLCO1B3*	Intron	c.85‐1847 T>G	‐	30% (21%, 40%)	1.4 × 10^−8^	0.26
Repaglinide AUC_0‐∞_	Step 1	rs4149117	*SLCO1B3*	Missense	c.334G>T	p.Ala112Ser	30% (21%, 40%)	1.5 × 10^−8^	0.26
Repaglinide AUC_0‐∞_	Step 1	rs7311358	*SLCO1B3*	Missense	c.699A>G	p.Ile233Met	30% (21%, 40%)	1.5 × 10^−8^	0.26
Repaglinide AUC_0‐∞_	Step 1	rs10841684	*SLCO1B3*	Intron	c.728‐3330A>T	‐	30% (21%, 40%)	1.5 × 10^−8^	0.26
Repaglinide AUC_0‐∞_	Step 1	rs11045797	*SLCO1B1*	Intron	c.84 + 15122 T>C	‐	−31% (−38%, −23%)	1.7 × 10^−8^	0.11
Repaglinide AUC_0‐∞_	Step 1	rs11045681	*SLCO1B3‐SLCO1B7*	Intron	c.1866‐20164A>G	‐	−30% (−37%, −23%)	3.7 × 10^−8^	0.12
Repaglinide AUC_0‐∞_	Step 1	rs11045676	*SLCO1B3‐SLCO1B7*	Intron	c.1866‐23829 T>A	‐	−30% (−37%, −23%)	4.4 × 10^−8^	0.12
Repaglinide AUC_0‐∞_	Step 2	rs11045916	*SLCO1A2*	3′UTR	c.*3042A>T	‐	−30% (−37%, −23%)	3.3 × 10^−9^	0.10
Repaglinide AUC_0‐∞_	Step 2	rs71446763	*SLCO1A2*	3′UTR	c.*4084C>T	‐	−30% (−36%, −22%)	9.5 × 10^−9^	0.10
Repaglinide AUC_0‐∞_	Step 2	rs11045917	*SLCO1A2*	3′UTR	c.*2534C>T	‐	−30% (−36%, −22%)	9.5 × 10^−9^	0.10
Repaglinide AUC_0‐∞_	Step 2	rs11045918	*SLCO1A2*	3′UTR	c.*1770G>T	‐	−30% (−36%, −22%)	9.5 × 10^−9^	0.10
Repaglinide AUC_0‐∞_	Step 2	rs6487215	*SLCO1A2*	Intron	c.1610 + 107C>T	‐	−30% (−36%, −22%)	9.5 × 10^−9^	0.10
Repaglinide AUC_0‐∞_	Step 2	rs10841795	*SLCO1A2*	Missense	c.38 T>C	Ile13Thr	−30% (−36%, −22%)	1.0 × 10^−8^	0.10
Repaglinide AUC_0‐∞_ during gemfibrozil	Step 1	rs972505	*SLCO1C1*	Intron	c.1799‐374C>A	‐	−21% (−25%, −16%)	1.1 × 10^−8^	0.48
Repaglinide AUC_0‐∞_ during gemfibrozil	Step 1	rs10841611	*SLCO1C1*	Intron	c.1916 + 31 T>C	‐	−21% (−25%, −16%)	1.1 × 10^−8^	0.48
Repaglinide AUC_0‐∞_ during gemfibrozil	Step 1	rs6487138	*SLCO1C1*	Synonymous	c.1927C>T	p.Leu643Leu	−20% (−25%, −16%)	1.6 × 10^−8^	0.48
Repaglinide AUC_0‐∞_ during gemfibrozil	Step 1	rs953001	*SLCO1C1*	3′UTR	c.*165C>T	‐	−20% (−25%, −16%)	1.6 × 10^−8^	0.48
Repaglinide AUC_0‐∞_ during gemfibrozil	Step 1	rs953002	*SLCO1C1*	3′UTR	c.*235C>T	‐	−20% (−25%, −16%)	1.6 × 10^−8^	0.48
Repaglinide AUC_0‐∞_ during gemfibrozil	Step 1	rs10444412	*SLCO1C1*	3′UTR	c.*528 T>C	‐	−20% (−25%, −16%)	1.6 × 10^−8^	0.48

Repaglinide AUC_0‐∝_ without concomitant medications was analyzed in 172 participants. Repaglinide AUC_0‐∞_ after gemfibrozil pretreatment was analyzed in 66 participants. Step 1 refers to the first part of the linear regression analysis, which included investigated variant with covariate, while in step 2 also the most significant variant of step 1 was included in the model.

AUC_0‐∞_, area under the plasma concentration–time curve from time zero to infinity; CI, confidence interval; MAF, minor‐allele frequency; rsID, reference SNP cluster ID.

In a GWAS of repaglinide AUC_0‐∞_ after gemfibrozil pretreatment, six nearly completely linked SNVs in the *SLCO1C1* gene associated with a decreased AUC_0‐∞_ of repaglinide (**Table** **1**, **Figure** **S2**). The strongest associations were observed with two completely linked (*r* ^2^ = 1, *D′* = 1) SNVs, rs10841611 and rs972505, and a decreased AUC_0‐∞_ of repaglinide (*P* = 1.1 × 10^−8^). The six *SLCO1C1* SNVs were not linked with the *SLCO1* SNVs associated with repaglinide AUC_0‐∞_ without concomitant medication or with any *SLCO1B1* missense variant. No genome‐wide significant associations were observed after accounting for *SLCO1C1* rs10841611. The fold increase in repaglinide AUC_0‐∞_ caused by gemfibrozil, gemfibrozil AUC, gemfibrozil 1‐O‐β‐glucuronide AUC, or gemfibrozil 1‐O‐β‐glucuronide/gemfibrozil AUC ratio showed no genome‐wide significant associations (**Figure** **S3**).

### Mechanistic studies of *SLCO1A2* and *SLCO1C1* associations

Since *SLCO1A2* SNVs associated strongly with a decreased AUC_0‐∞_ of repaglinide, repaglinide transport by OATP1A2 was investigated *in vitro*; however, MDCKII cells overexpressing OATP1A2 showed no significant uptake of repaglinide (**Figure** **S4**). *SLCO1C1*, which showed genome‐wide significant associations with repaglinide pharmacokinetics, has not been previously reported to influence pharmacokinetics. Because *SLCO1C1* is located near *SLCO1B1* and *SLCO1B3*, which both transport repaglinide, associations of *SLCO1C1* rs10841611 were investigated in two human liver sample cohorts. In the first cohort, *SLCO1C1* rs10841611 showed no significant associations with OATP1B1 or OATP1B3 protein expression nor with mRNA expression of *SLCO1B1*, *SLCO1B3*, or *SLCO1C1* (**Figure** **S5**). In the second cohort, the expression of *SLCO1B3* main transcript (NM_019844.4) was 18% (90% CI: 6–31%; *P* = 0.038) and 33% (90% CI: 18–51%; *P* = 3.6 × 10.^4^) higher in *SLCO1C1* rs10841611 heterozygotes and homozygotes, respectively, than in noncarriers (**Figure** **S5**). Furthermore, we investigated the associations of *SLCO1C1* rs10841611 with the OATP1B1 biomarker GCDCA‐3G and the OATP1B3 biomarker GCDCA‐S in healthy participants, but *SLCO1C1* rs10841611 showed no significant associations with these compounds (**Table** **S6**). *SLCO1C1* rs10841611 did not associate with the AUC of gemfibrozil or gemfibrozil 1‐O‐β‐glucuronide (**Table** **S7**).

### Effects of *SLCO1B1* , *CYP2C8* , and *SLCO1C1* genotypes on repaglinide pharmacokinetics without concomitant medication

Based on the above findings, we next investigated the effects of *SLCO1B1*, *CYP2C8*, and *SLCO1C1* genotype groups on repaglinide pharmacokinetics. The AUC_0‐∞_ of repaglinide without concomitant medication was 105% (90% CI: 74–143%; *P* = 8.7 × 10^−11^) and 22% (90% CI: 7–38%; *P* = 0.011) greater in participants with the poor and decreased function *SLCO1B1* genotypes, respectively, than in those with the normal function *SLCO1B1* genotype (**Table** **2**, **Figure** **S6**). Conversely, the AUC_0‐∞_ was 26% (90% CI: 7–42%; *P* = 0.034) and 21% (90% CI: 9–31%; *P* = 0.0055) lower in participants with the highly increased and increased function *SLCO1B1* genotypes, respectively, than in those with the normal function *SLCO1B1* genotype. In participants with the CYP2C8*19/*19 genotype, the AUC_0‐∞_ of repaglinide was 45% (90% CI: 24–70%; *P* = 1.6 × 10^−4^) greater than in participants with the CYP2C8*1/*1 genotype. Furthermore, the AUC_0‐∞_ of repaglinide was 26% (90% CI: 13–37%; *P* = 0.0033) smaller in participants with the CYP2C8*1/*3 genotype and 51% (90% CI: 24–84%; *P* = 8.2 × 10^−4^) greater in those with the CYP2C8*1/*4 genotype, than in participants with the CYP2C8*1/*1 genotype. Compared with *SLCO1C1* rs10841611 noncarriers, the AUC_0‐∞_ of repaglinide was 18% (90% CI: 7–27%; *P* = 0.01) and 26% (90% CI: 15–35%; *P* = 4.7 × 10^−4^) lower in *SLCO1C1* rs10841611 heterozygotes and homozygotes, respectively.

**Table 2:** Associations of *SLCO1B1* genotype groups and *CYP2C8* and *SLCO1C1* genotypes with the AUC_0‐∞_ of repaglinide (*n* = 166) without concomitant medication

Trait	Genotype group (*n*, %)	Geometric mean (90% CI)	GMR (90% CI)	*P*
Repaglinide AUC_0‐∞_ (ng × h/mL)	*SLCO1B1*			
	Highly increased function (5, 3%)	3.2 (2.5, 3.9)	0.74 (0.58, 0.93)	0.034
	Increased function (30, 18%)	3.4 (3.0, 3.8)	0.79 (0.69, 0.91)	0.0055
	Normal function (64, 39%)	4.3 (4.0, 4.6)	1	
	Decreased function (50, 30%)	5.2 (4.7, 5.7)	1.22 (1.07, 1.38)	0.011
	Poor function (17, 10%)	8.8 (7.6, 10.2)	2.05 (1.74, 2.43)	8.7 × 10^−11^
	*CYP2C8*			
	*1/*3 (15, 9%)	3.1 (2.7, 3.6)	0.74 (0.63, 0.87)	0.0033
	*3/*19 (3, 2%)	3.2 (2.4, 4.3)	0.76 (0.57, 1.03)	0.13
	*1/*1 (56, 34%)	4.2 (3.9, 4.6)	1	
	*1/*19 (55, 33%)	4.7 (4.3, 5.2)	1.13 (0.99, 1.28)	0.13
	*1/*4 (10, 6%)	6.4 (5.3, 7.6)	1.51 (1.24, 1.84)	8.2 × 10^−4^
	*4/*19 (5, 3%)	4.5 (3.6, 5.7)	1.07 (0.84, 1.37)	0.65
	*19/*19 (22, 13%)	6.1 (5.4, 7.0)	1.45 (1.24, 1.70)	1.6 × 10^−4^
	*SLCO1C1* rs10841611 T>C			
	Noncarrier (42, 25%)	5.5 (5.0, 6.0)	1	
	Heterozygote (86, 52%)	4.5 (4.2, 4.9)	0.82 (0.73, 0.93)	0.010
	Homozygote (38, 23%)	4.1 (3.7, 4.5)	0.74 (0.65, 0.85)	4.7 × 10^−4^

Note: The data are estimated geometric marginal means from a general linear model analysis. *SLCO1B1* results are adjusted for *CYP2C8* and *SLCO1C1*, *CYP2C8* results are adjusted for *SLCO1B1* and *SLCO1C1*, and *SLCO1C1* results are adjusted for *SLCO1B1* and *CYP2C8*. Two participants had *SLCO1B1* genotype with undefined function and they were excluded from the analysis. CYP2C8*3/*3 and CYP2C8*4/*4 genotypes were observed in only two participants, and therefore, they were excluded from the analysis.

AUC_0–∞_, area under the plasma concentration–time curve from time zero to infinity; CI, confidence interval; GMR, geometric mean ratio to reference group.

### Effects of *SLCO1B1* , *CYP2C8* , and *SLCO1C1* genotypes on the gemfibrozil–repaglinide interaction

**Figure 4:**
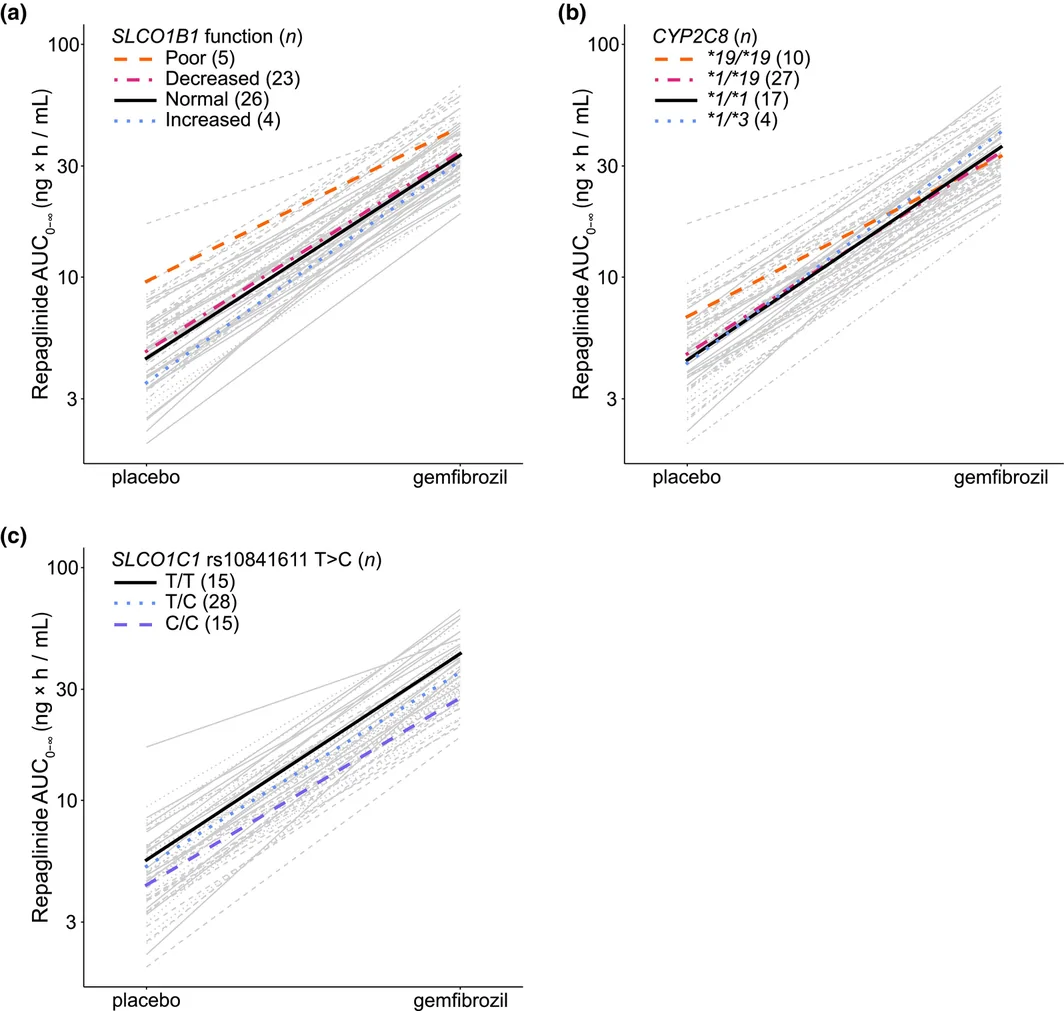
Effects of *SLCO1B1*, *CYP2C8*, and *SLCO1C1* genotypes on the gemfibrozil–repaglinide interaction. Individual repaglinide AUC_0‐∞_ values during the placebo and gemfibrozil phases are connected with gray lines (*n* = 58). Colored lines indicate geometric means of repaglinide AUC_0‐∞_ in the different genotype groups.

The AUC_0‐∞_ of repaglinide after gemfibrozil pretreatment was 35% (90% CI: 24–45%; *P* = 5.6 × 10^−5^) and 17% (90% CI: 5–27%; *P* = 0.029) lower in *SLCO1C1* rs10841611 homozygotes and heterozygotes, respectively, than in *SLCO1C1* rs10841611 noncarriers (**Table** **3**, **Figure** **S6**). The AUC_0‐∞_ of repaglinide after gemfibrozil pretreatment was 32% (90% CI: 9–61%; *P* = 0.020) greater in participants with the poor function *SLCO1B1* genotype than in participants with the normal function *SLCO1B1* genotype. The fold increase in repaglinide AUC_0‐∞_ caused by gemfibrozil was 36% (90% CI: 24–47%; *P* = 1.3 × 10^−4^) lower in participants with the CYP2C8*19/*19 genotype than in those with the CYP2C8*1/*1 genotype (**Table** **3**, **Figure** **4**). Compared with participants with the normal function *SLCO1B1* genotype, the fold increase in repaglinide AUC_0‐∞_ by gemfibrozil was 32% (90% CI: 16–45%; *P* = 0.0045) lower in participants with the poor function *SLCO1B1* genotype.

**Table 3:** Associations of *SLCO1B1* genotype groups and *CYP2C8* and *SLCO1C1* genotypes with the pharmacokinetics of repaglinide (*n* = 58) after gemfibrozil pretreatment

Trait	Genotype group (*n*, %)	Geometric mean (90% CI)	GMR (90% CI)	*P*
Repaglinide AUC_0‐∞_ after gemfibrozil pretreatment (ng × h/mL)	*SLCO1B1*			
	Increased function (4, 7%)	31.7 (26.2, 38.3)	0.95 (0.77, 1.17)	0.67
	Normal function (26, 45%)	33.4 (30.7, 36.3)	1	
	Decreased function (23, 40%)	34.5 (31.6, 37.8)	1.03 (0.91, 1.17)	0.64
	Poor function (5, 9%)	44.2 (37.1, 52.6)	1.32 (1.09, 1.61)	0.020
	*CYP2C8*			
	*1/*3 (4, 7%)	42.0 (35.2, 50.1)	1.16 (0.94, 1.42)	0.23
	*1/*1 (17, 29%)	36.2 (32.7, 40.1)	1	
	*1/*19 (27, 47%)	34.5 (31.4, 37.8)	0.95 (0.83, 1.09)	0.55
	*19/*19 (10, 17%)	33.0 (29.3, 37.3)	0.91 (0.78, 1.07)	0.34
	*SLCO1C1* rs10841611 T>C			
	Noncarrier (15, 26%)	42.7 (38.5, 47.3)	1	
	Heterozygote (28, 48%)	35.6 (32.6, 38.7)	0.83 (0.73, 0.95)	0.029
	Homozygote (15, 26%)	27.5 (24.5, 31.0)	0.65 (0.55, 0.76)	5.6 × 10^−5^
Fold increase in repaglinide AUC_0‐∞_ caused by gemfibrozil	*SLCO1B1*			
	Increased function (4, 7%)	8.4 (6.7, 10.4)	1.12 (0.88, 1.43)	0.42
	Normal function (26, 45%)	7.5 (6.8, 8.2)	1	
	Decreased function (23, 40%)	7.1 (6.4, 7.9)	0.95 (0.83, 1.10)	0.58
	Poor function (5, 9%)	5.1 (4.2, 6.1)	0.68 (0.55, 0.84)	0.0045
	*CYP2C8*			
	*1/*3 (4, 7%)	10.1 (8.2, 12.4)	1.26 (1.00, 1.60)	0.10
	*1/*1 (17, 29%)	8.0 (7.1, 8.9)	1	
	*1/*19 (27, 47%)	7.4 (6.6, 8.3)	0.93 (0.79, 1.09)	0.45
	*19/*19 (10, 17%)	5.1 (4.4, 5.8)	0.64 (0.53, 0.76)	1.3 × 10^−4^
	*SLCO1C1* rs10841611 T>C			
	Noncarrier (15, 26%)	8.0 (7.1, 8.9)	1	
	Heterozygote (28, 48%)	6.7 (6.1, 7.4)	0.84 (0.72, 0.98)	0.059
	Homozygote (15, 26%)	6.4 (5.6, 7.4)	0.81 (0.67, 0.97)	0.054

The data are estimated geometric marginal means from a general linear model analysis. *SLCO1B1* results are adjusted for *CYP2C8* and *SLCO1C1*, *CYP2C8* results are adjusted for *SLCO1B1* and *SLCO1C1*, and *SLCO1C1* results are adjusted for *SLCO1B1* and *CYP2C8*. Only two participants had highly increased function *SLCO1B1* genotype or CYP2C8*3/*19 genotype, thus they were excluded from the analyses. Each of the CYP2C8*1/*4, CYP2C8*3/*3, CYP2C8*4/*4, or CYP2C8*4/*19 genotypes was observed in only one participant, and therefore, those participants were excluded from the analyses.

AUC_0–∞_, area under the plasma concentration–time curve from time zero to infinity; CI, confidence interval; GMR, geometric mean ratio to reference group.

## DISCUSSION

This study investigated pharmacogenetic variation of repaglinide and gemfibrozil disposition, as well as their interaction. We identified a novel functional *CYP2C8* allele, CYP2C8*19, which was associated with increased AUC_0‐∞_ of repaglinide and another CYP2C8 substrate, montelukast. CYP2C8*19 is defined by the rs2071426 variant, which introduces an additional splice site, leading to transcript‐specific alterations in *CYP2C8* mRNA expression and reduced CYP2C8 protein expression and activity. In the GWAS of repaglinide without concomitant medication, the *SLCO1B1* c.521 T>C no function allele showed a strong association with increased AUC_0‐∞_ of repaglinide, whereas *SLCO1A2* variants were associated with decreased repaglinide AUC_0‐∞_. However, as OATP1A2 did not transport repaglinide *in vitro*, the observed *SLCO1A2* associations are likely attributable to linkage disequilibrium with the increased function SLCO1B1*14 allele. Additionally, six *SLCO1C1* variants showed genome‐wide significant associations with repaglinide AUC_0‐∞_ after gemfibrozil pretreatment.

The CYP2C8*19 defining variant (rs2071426) is located in intron 1 of *CYP2C8* and creates a splice donor site, resulting in the inclusion of an additional exon through alternative splicing. In human liver samples, CYP2C8*19 associated with increased expression of a transcript with the additional exon (NM_001198855.1) and reduced expression of the main protein‐coding transcript (NM_000770.3), resulting in decreased CYP2C8 protein expression and enzyme activity. Consistent with our results, splicing quantitative trait locus data from the Genotype‐Tissue Expression project shows a strong association of rs2071426 with reduced splicing of the first intron of *CYP2C8* in the liver (*P* = 6.8 × 10^−33^).^41^ The alternatively spliced NM_001198855.1 encodes a protein (NP_001185784.1) that is 70 amino acids shorter than the canonical isoform, resulting in the loss of the N‐terminal α‐helix responsible for anchoring the enzyme to the endoplasmic reticulum.^42^ The CYP2C8*19 allele had a moderate effect on repaglinide pharmacokinetics, as CYP2C8*19 homozygotes had 45% greater repaglinide AUC_0‐∞_ than noncarriers, consistent with a 33% decrease in repaglinide clearance. This is the first study that describes the CYP2C8*19 allele, shows that it influences the pharmacokinetics of CYP2C8 substrates, and provides a mechanistic explanation for the associations.

The strong association of *SLCO1B1* c.521 T>C with increased repaglinide AUC_0‐∞_ observed in the GWAS is consistent with previous studies^8^, ^23^, ^25^, ^26^ and is explained by loss of OATP1B1 function.^8^, ^9^ Although *SLCO1A2* variants associated with decreased repaglinide AUC_0‐∞_, OATP1A2 did not transport repaglinide *in vitro*. Therefore, a likely explanation is linkage disequilibrium with the increased function SLCO1B1*14 allele, which increases hepatic OATP1B1 expression.^39^, ^43^ The mechanism behind increased OATP1B1 expression remains unclear, but it may involve altered microRNA binding in the *SLCO1B1* 3′UTR, as multiple 3′UTR variants are present in the SLCO1B1*14 haplotype (**Figure** **S7**).

Associations between *SLCO1C1* variants and repaglinide AUC_0‐∞_ have not been reported previously, nor has *SLCO1C1* been implicated in the pharmacokinetics of other drugs. *SLCO1C1* is predominantly expressed in the brain and pituitary gland, unlike the liver‐enriched *SLCO1B1* and *SLCO1B3*.^44^, ^45^ The *SLCO1C1* variants were not in linkage disequilibrium with known functional variants of the nearby *SLCO1B1* and showed no associations with OATP1B1 or OATP1B3 biomarker levels. While *SLCO1C1* rs10841611 associated with slightly increased *SLCO1B3* mRNA expression in one liver cohort, no corresponding effects on protein expression were observed. Moreover, gemfibrozil did not modify the impact of *SLCO1C1* variants on repaglinide pharmacokinetics, supporting an OATP1B1‐independent mechanism. Further studies are needed to elucidate the role of *SLCO1C1* in pharmacokinetics and the underlying biological mechanisms.

In addition to CYP2C8*19, the CYP2C8*3 and CYP2C8*4 alleles associated with repaglinide pharmacokinetics. Similar to previous studies with repaglinide, montelukast, and cinitapride, the CYP2C8*3 allele associated with decreased exposure to repaglinide, consistent with increased metabolic clearance.^3^, ^8^, ^36^, ^46^ The mechanism behind increased enzyme activity related to CYP2C8*3 is unclear and for some other drugs unchanged or decreased metabolism has also been reported suggesting a substrate specific effect.^3^, ^47^, ^48^ Opposite to CYP2C8*3, CYP2C8*4 associated with a slightly increased AUC_0‐∞_ of repaglinide, consistent with the results of previous studies with other CYP2C8 substrates.^36^, ^46^ Our data suggest that CYP2C8*3 increases and CYP2C8*4 decreases repaglinide metabolism, but it is important to keep in mind that the effects may be substrate specific.

Gemfibrozil increased repaglinide AUC_0‐∞_ only fivefold in participants with the CYP2C8*19/*19 genotype, while the average increase was 7.6‐fold. This can be explained by a smaller fraction of repaglinide metabolized by CYP2C8 in participants with the CYP2C8*19/*19 genotype than in those with CYP2C8*1/*1. Similarly, gemfibrozil increased repaglinide AUC_0‐∞_ fivefold in participants with the poor function *SLCO1B1* genotype. Because these participants lack OATP1B1 activity, this effect largely reflects the contribution of CYP2C8 inhibition to the gemfibrozil–repaglinide interaction. The finding that gemfibrozil increased repaglinide AUC_0‐∞_ by approximately one‐third less in participants with the poor function *SLCO1B1* genotype than in those with the normal function genotype suggests that gemfibrozil inhibits OATP1B1 activity by ~ 50%.

This study demonstrates that reduced‐function *CYP2C8* and *SLCO1B1* genotypes may lead to underestimation of CYP2C8‐ or OATP1B1‐mediated drug–drug‐interactions with repaglinide. The ICH M12 guideline recommends excluding individuals with poor function genotypes or evaluating drug–drug interactions separately by genotype when investigating substrates or inhibitors of polymorphic enzymes or transporters.^1^ Although CYP2C8*19/*19 is only a decreased function genotype, the magnitude of the gemfibrozil–repaglinide interaction was considerably smaller in participants with this genotype. In contrast, the interaction appeared greater in CYP2C8*3 carriers, but this association was not statistically significant, likely due to the small number of CYP2C8*3 carriers. The relatively high frequency of the CYP2C8*19/*19 genotype supports genotyping of participants in repaglinide interaction studies to enable stratification of results.

No significant associations were observed in the gemfibrozil GWAS. Although the sample size was relatively large for a pharmacokinetic study, it was likely underpowered to detect rare variants or variants with small effects. The data originated from drug–drug interaction studies with gemfibrozil as the perpetrator, but the victim drugs are not known to affect gemfibrozil pharmacokinetics, and variability in gemfibrozil pharmacokinetics was low between studies. Overall, this study suggests the absence of common genetic variants with large effects on gemfibrozil pharmacokinetics, while effects of rare variants cannot be excluded.

This study included only white Finnish participants. Allele frequencies of pharmacogenetic variants vary across populations. Although the functional impact of these variants on repaglinide pharmacokinetics is expected to be consistent across populations, the proportion of variability attributable to each variant depends on its population‐specific frequency. In the Finnish population, the CYP2C8*19 allele was the most prevalent *CYP2C8* variant allele, with a frequency of 28%. The frequency of CYP2C8*19 is similar among European and South Asian populations, but notably lower in East Asians and Africans (**Table** **S8**). In sub‐Saharan African and African American populations, rs2071426 is strongly linked (*r* ^2^ = 0.61–0.79, *D′* = 0.97–1.00) with the *CYP2C8* c.805A>T missense variant (rs11572103, p.Ile269Phe), defining the CYP2C8*2 allele (**Table** **S8**).^49^ In one study, CYP2C8*2 associated with decreased metabolism of the CYP2C8 substrate pioglitazone in African Americans.^50^ Further studies are needed to determine if rs2071426 contributes to the reduced activity of CYP2C8*2.

In conclusion, these data indicate that genetic variation in *SLCO1* and *CYP2C8* play important roles in the pharmacokinetics of repaglinide and in explaining interindividual variability in the gemfibrozil–repaglinide interaction. Moreover, we characterized a novel *CYP2C8* allele, CYP2C8*19, which is relatively frequent and results in an alternative *CYP2C8* transcript, reduces the protein expression of CYP2C8, and increases the exposure to CYP2C8 substrates. Our data also demonstrated previously unidentified genome‐wide significant associations of *SLCO1C1* variants with repaglinide pharmacokinetics after gemfibrozil pretreatment. The mechanisms of these associations remain unclear but appear to be independent of OATP1B1 and OATP1B3. Overall, the results indicate that pharmacogenetic variation should be considered in the interpretation of drug–drug interactions studies.

## FUNDING

This study was supported by grants from the Sigrid Jusélius Foundation (Helsinki, Finland) and State funding for university‐level health research (Helsinki University Hospital, Finland). MS and KK were in part supported by the Robert Bosch Stiftung Stuttgart, Germany. JIK was supported by the Paulo Foundation (Helsinki, Finland) and the Finnish Cultural Foundation (Helsinki, Finland). DK was supported by the Research Council of Finland (contract 316458). The Kuopio Obesity Surgery Study was supported by the Finnish Diabetes Research Foundation (Tampere, Finland), State funding for university‐level health research (Kuopio University Hospital, Finland), the Research Council of Finland (grant no. 138006), the Finnish Cultural Foundation, and the University of Eastern Finland Spearhead Funding.

## CONFLICT OF INTEREST

The authors declared no competing interests for this work.

## AUTHOR CONTRIBUTIONS

A.J.H.M., P.H., and M.Ni. wrote the manuscript; A.J.H.M. and M.Ni. designed the research; A.J.H.M., P.H., K.K., D.K., M.G., T.T., J.I.K., M.Ne., V.T.M., J.P., M.T., M.S., A.T., J.T.B., and M.Ni. performed the research; A.J.H.M. and M.Ni. analyzed the data.

## Supporting information


Data S1.

## References

[1] International Council for Harmonisation of Technical Requirement for Pharmaceuticals for Human Use . Drug Interaction Studies M12 (2024) <https://database.ich.org/sites/default/files/ICH_M12_Step4_Guideline_2024_0521_0.pdf>.

[2] Bidstrup, T.B. , Bjørnsdottir, I. , Sidelmann, U.G. , Thomsen, M.S. & Hansen, K.T. CYP2C8 and CYP3A4 are the principal enzymes involved in the human in vitro biotransformation of the insulin secretagogue repaglinide. Br. J. Clin. Pharmacol. 56, 305–314 (2003). 12919179 10.1046/j.0306-5251.2003.01862.x PMC1884358

[3] Backman, J.T. , Filppula, A.M. , Niemi, M. & Neuvonen, P.J. Role of cytochrome P450 2C8 in drug metabolism and interactions. Pharmacol. Rev. 68, 168–241 (2016). 26721703 10.1124/pr.115.011411

[4] Niemi, M. , Backman, J.T. , Neuvonen, M. & Neuvonen, P.J. Effects of gemfibrozil, itraconazole, and their combination on the pharmacokinetics and pharmacodynamics of repaglinide: potentially hazardous interaction between gemfibrozil and repaglinide. Diabetologia 46, 347–351 (2003). 12687332 10.1007/s00125-003-1034-7

[5] Niemi, M. , Kajosaari, L.I. , Neuvonen, M. , Backman, J.T. & Neuvonen, P.J. The CYP2C8 inhibitor trimethoprim increases the plasma concentrations of repaglinide in healthy subjects. Br. J. Clin. Pharmacol. 57, 441–447 (2004). 15025742 10.1046/j.1365-2125.2003.02027.x PMC1884466

[6] Kajosaari, L.I. , Niemi, M. , Neuvonen, M. , Laitila, J. , Neuvonen, P.J. & Backman, J.T. Cyclosporine markedly raises the plasma concentrations of repaglinide. Clin. Pharmacol. Ther. 78, 388–399 (2005). 16198658 10.1016/j.clpt.2005.07.005

[7] Kajosaari, L.I. , Niemi, M. , Backman, J.T. & Neuvonen, P.J. Telithromycin, but not montelukast, increases the plasma concentrations and effects of the cytochrome P450 3A4 and 2C8 substrate repaglinide. Clin. Pharmacol. Ther. 79, 231–242 (2006). 16513447 10.1016/j.clpt.2005.11.002

[8] Niemi, M. *et al*. Polymorphic organic anion transporting polypeptide 1B1 is a major determinant of repaglinide pharmacokinetics. Clin. Pharmacol. Ther. 77, 468–478 (2005). 15961978 10.1016/j.clpt.2005.01.018

[9] Varma, M.V.S. , Lai, Y. , Kimoto, E. , Goosen, T.C. , el‐Kattan, A.F. & Kumar, V. Mechanistic modeling to predict the transporter‐ and enzyme‐mediated drug‐drug interactions of repaglinide. Pharm. Res. 30, 1188–1199 (2013). 23307347 10.1007/s11095-012-0956-5

[10] Backman, J.T. *et al*. CYP2C8 activity recovers within 96 hours after gemfibrozil dosing: estimation of CYP2C8 half‐life using repaglinide as an in vivo probe. Drug Metab. Dispos. 37, 2359–2366 (2009). 19773535 10.1124/dmd.109.029728

[11] Honkalammi, J. , Niemi, M. , Neuvonen, P.J. & Backman, J.T. Dose‐dependent interaction between gemfibrozil and repaglinide in humans: strong inhibition of CYP2C8 with subtherapeutic gemfibrozil doses. Drug Metab. Dispos. 39, 1977–1986 (2011). 21778352 10.1124/dmd.111.040931

[12] Honkalammi, J. , Niemi, M. , Neuvonen, P.J. & Backman, J.T. Mechanism‐based inactivation of CYP2C8 by gemfibrozil occurs rapidly in humans. Clin. Pharmacol. Ther. 89, 579–586 (2011). 21368757 10.1038/clpt.2010.358

[13] Karonen, T. , Filppula, A. , Laitila, J. , Niemi, M. , Neuvonen, P.J. & Backman, J.T. Gemfibrozil markedly increases the plasma concentrations of montelukast: a previously unrecognized role for CYP2C8 in the metabolism of montelukast. Clin. Pharmacol. Ther. 88, 223–230 (2010). 20592724 10.1038/clpt.2010.73

[14] Karonen, T. , Neuvonen, P.J. & Backman, J.T. CYP2C8 but not CYP3A4 is important in the pharmacokinetics of montelukast. Br. J. Clin. Pharmacol. 73, 257–267 (2012). 21838784 10.1111/j.1365-2125.2011.04086.x PMC3269585

[15] Filppula, A.M. , Tornio, A. , Niemi, M. , Neuvonen, P.J. & Backman, J.T. Gemfibrozil impairs imatinib absorption and inhibits the CYP2C8‐mediated formation of its main metabolite. Clin. Pharmacol. Ther. 94, 383–393 (2013). 23657159 10.1038/clpt.2013.92

[16] Backman, J.T. , Luurila, H. , Neuvonen, M. & Neuvonen, P.J. Rifampin markedly decreases and gemfibrozil increases the plasma concentrations of atorvastatin and its metabolites. Clin. Pharmacol. Ther. 78, 154–167 (2005). 16084850 10.1016/j.clpt.2005.04.007

[17] Tornio, A. , Niemi, M. , Neuvonen, P.J. & Backman, J.T. Stereoselective interaction between the CYP2C8 inhibitor gemfibrozil and racemic ibuprofen. Eur. J. Clin. Pharmacol. 63, 463–469 (2007). 17333159 10.1007/s00228-007-0273-9

[18] Niemi, M. , Tornio, A. , Pasanen, M.K. , Fredrikson, H. , Neuvonen, P.J. & Backman, J.T. Itraconazole, gemfibrozil and their combination markedly raise the plasma concentrations of loperamide. Eur. J. Clin. Pharmacol. 62, 463–472 (2006). 16758263 10.1007/s00228-006-0133-z

[19] Niemi, M. , Backman, J.T. , Granfors, M. , Laitila, J. , Neuvonen, M. & Neuvonen, P.J. Gemfibrozil considerably increases the plasma concentrations of rosiglitazone. Diabetologia 46, 1319–1323 (2003). 12898007 10.1007/s00125-003-1181-x

[20] Tornio, A. *et al*. Glucuronidation converts clopidogrel to a strong time‐dependent inhibitor of CYP2C8: a phase II metabolite as a perpetrator of drug‐drug interactions. Clin. Pharmacol. Ther. 96, 498–507 (2014). 24971633 10.1038/clpt.2014.141

[21] Aurinsalo, L. *et al*. A phenotyping tool for seven cytochrome P450 enzymes and two transporters: application to examine the effects of Clopidogrel and gemfibrozil. Clin. Pharmacol. Ther. 117, 1732–1742 (2025). 39982209 10.1002/cpt.3610 PMC12087695

[22] Tornio, A. *et al*. The effect of gemfibrozil on repaglinide pharmacokinetics persists for at least 12 h after the dose: evidence for mechanism‐based inhibition of CYP2C8 in vivo. Clin. Pharmacol. Ther. 84, 403–411 (2008). 18388877 10.1038/clpt.2008.34

[23] Kalliokoski, A. , Backman, J.T. , Kurkinen, K.J. , Neuvonen, P.J. & Niemi, M. Effects of gemfibrozil and atorvastatin on the pharmacokinetics of repaglinide in relation to SLCO1B1 polymorphism. Clin. Pharmacol. Ther. 84, 488–496 (2008). 19238654 10.1038/clpt.2008.74

[24] Honkalammi, J. , Niemi, M. , Neuvonen, P.J. & Backman, J.T. Gemfibrozil is a strong inactivator of CYP2C8 in very small multiple doses. Clin. Pharmacol. Ther. 91, 846–855 (2012). 22472994 10.1038/clpt.2011.313

[25] Kalliokoski, A. , Neuvonen, M. , Neuvonen, P.J. & Niemi, M. Different effects of SLCO1B1 polymorphism on the pharmacokinetics and pharmacodynamics of repaglinide and nateglinide. J. Clin. Pharmacol. 48, 311–321 (2008). 18187595 10.1177/0091270007311569

[26] Kalliokoski, A. , Neuvonen, M. , Neuvonen, P.J. & Niemi, M. The effect of SLCO1B1 polymorphism on repaglinide pharmacokinetics persists over a wide dose range. Br. J. Clin. Pharmacol. 66, 818–825 (2008). 18823304 10.1111/j.1365-2125.2008.03287.x PMC2675779

[27] Niemi, M. , Leathart, J.B. , Neuvonen, M. , Backman, J.T. , Daly, A.K. & Neuvonen, P.J. Polymorphism in CYP2C8 is associated with reduced plasma concentrations of repaglinide. Clin. Pharmacol. Ther. 74, 380–387 (2003). 14534525 10.1016/S0009-9236(03)00228-5

[28] Stephens, M. , Smith, N.J. & Donnelly, P. A new statistical method for haplotype reconstruction from population data. Am. J. Hum. Genet. 68, 978–989 (2001). 11254454 10.1086/319501 PMC1275651

[29] Stephens, M. & Scheet, P. Accounting for decay of linkage disequilibrium in haplotype inference and missing‐data imputation. Am. J. Hum. Genet. 76, 449–462 (2005). 15700229 10.1086/428594 PMC1196397

[30] Gaedigk, A. , Casey, S.T. , Whirl‐Carrillo, M. , Miller, N.A. & Klein, T.E. Pharmacogene variation consortium: a global resource and repository for Pharmacogene variation. Clin. Pharmacol. Ther. 110, 542–545 (2021). 34091888 10.1002/cpt.2321 PMC8725060

[31] Neuvonen, M. , Tornio, A. , Hirvensalo, P. , Backman, J.T. & Niemi, M. Performance of plasma coproporphyrin I and III as OATP1B1 biomarkers in humans. Clin. Pharmacol. Ther. 110, 1622–1632 (2021). 34580865 10.1002/cpt.2429 PMC9292572

[32] Hubeny, A. *et al*. Expression of organic anion transporting polypeptide 1A2 in red blood cells and its potential impact on antimalarial therapy. Drug Metab. Dispos. 44, 1562–1568 (2016). 27504015 10.1124/dmd.116.069807

[33] Neuvonen, M. *et al*. Identification of glycochenodeoxycholate 3‐O‐glucuronide and glycodeoxycholate 3‐O‐glucuronide as highly sensitive and specific OATP1B1 biomarkers. Clin. Pharmacol. Ther. 109, 646–657 (2021). 32961594 10.1002/cpt.2053 PMC7983942

[34] Orozco, C.C. *et al*. Characterization of bile acid sulfate conjugates as substrates of human organic anion transporting polypeptides. Mol. Pharm. 20, 3020–3032 (2023). 37134201 10.1021/acs.molpharmaceut.3c00040

[35] Männistö, V. *et al*. Protein phosphatase 1 regulatory subunit 3B genotype at rs4240624 has a major effect on gallbladder bile composition. Hepatol. Commun. 5, 244–257 (2021). 33553972 10.1002/hep4.1630 PMC7850313

[36] Hirvensalo, P. *et al*. Comprehensive Pharmacogenomic study reveals an important role of UGT1A3 in Montelukast pharmacokinetics. Clin. Pharmacol. Ther. 104, 158–168 (2018). 28940478 10.1002/cpt.891 PMC6033076

[37] Thomas, M. *et al*. Peroxisome proliferator‐activated receptor alpha, PPARα, directly regulates transcription of cytochrome P450 CYP2C8. Front. Pharmacol. 6, 261 (2015). 26582990 10.3389/fphar.2015.00261 PMC4631943

[38] Weiß, F. *et al*. Direct quantification of cytochromes P450 and drug transporters‐a rapid, targeted mass spectrometry‐based immunoassay panel for tissues and cell culture lysates. Drug Metab. Dispos. 46, 387–396 (2018). 29343608 10.1124/dmd.117.078626

[39] Nies, A.T. *et al*. Genetics is a major determinant of expression of the human hepatic uptake transporter OATP1B1, but not of OATP1B3 and OATP2B1. Genome Med. 5, 1 (2013). 23311897 10.1186/gm405 PMC3706890

[40] Rangwala, S.H. *et al*. Accessing NCBI data using the NCBI sequence viewer and genome data viewer (GDV). Genome Res. 31, 159–169 (2021). 33239395 10.1101/gr.266932.120 PMC7849379

[41] The GTEx consortium atlas of genetic regulatory effects across human tissues. Science 369, 1318–1330 (2020). 32913098 10.1126/science.aaz1776 PMC7737656

[42] Estrada, D.F. , Kumar, A. , Campomizzi, C.S. & Jay, N. Crystal structures of drug‐metabolizing CYPs. Methods Mol. Biol. 2342, 171–192 (2021). 34272695 10.1007/978-1-0716-1554-6_7 PMC10813703

[43] Prasad, B. *et al*. Interindividual variability in hepatic organic anion‐transporting polypeptides and P‐glycoprotein (ABCB1) protein expression: quantification by liquid chromatography tandem mass spectroscopy and influence of genotype, age, and sex. Drug Metab. Dispos. 42, 78–88 (2014). 24122874 10.1124/dmd.113.053819 PMC3876790

[44] Uhlén, M. *et al*. Proteomics. Tissue‐based map of the human proteome. Science 347, 1260419 (2015). 25613900 10.1126/science.1260419

[45] The Human Protein Atlas <https://www.proteinatlas.org/>.

[46] Campodónico, D.M. *et al*. CYP2C8*3 and *4 define CYP2C8 phenotype: an approach with the substrate cinitapride. Clin. Transl. Sci. 15, 2613–2624 (2022). 36065758 10.1111/cts.13386 PMC9652446

[47] Marcath, L.A. *et al*. Patients carrying CYP2C8*3 have shorter systemic paclitaxel exposure. Pharmacogenomics 20, 95–104 (2019). 30520341 10.2217/pgs-2018-0162 PMC6562943

[48] Rowbotham, S.E. , Boddy, A.V. , Redfern, C.P.F. , Veal, G.J. & Daly, A.K. Relevance of nonsynonymous CYP2C8 polymorphisms to 13‐*cis* retinoic acid and paclitaxel hydroxylation. Drug Metab. Dispos. 38, 1261–1266 (2010). 20421446 10.1124/dmd.109.030866

[49] Auton, A. *et al*. A global reference for human genetic variation. Nature 526, 68–74 (2015). 26432245 10.1038/nature15393 PMC4750478

[50] Aquilante, C.L. , Wempe, M.F. , Spencer, S.H. , Kosmiski, L.A. , Predhomme, J.A. & Sidhom, M.S. Influence of CYP2C8*2 on the pharmacokinetics of pioglitazone in healthy African‐American volunteers. Pharmacotherapy 33, 1000–1007 (2013). 23712614 10.1002/phar.1292 PMC3760990

