# Supplementary material for: Identification of a Functional CYP2C8 Variant Allele that Alters Splicing, Reduces Protein Expression, and Increases Drug Exposure
Source: Clin Pharmacol Ther. 2026 Jul 1:10.1002/cpt.70376. Online ahead of print. doi: 10.1002/cpt.70376 (PMC13338972; doi:10.1002/cpt.70376)
Supplement: Supplementary file 1 — Data S1. [file CPT-9999-0-s001.pdf]

**Table S1** Details of original pharmacokinetic studies.

| Original study reference | GWAS (*n*) Repaglinide no concomitant mediation | Repaglinide after gemfibrozil pretreatment | Gemfibrozil | Gemfibrozil 1-O-β glucuronide | Repaglinide dose (mg) | Gemfibrozil dose (mg) | Total number gemfibrozil doses (b.i.d.) |
|---|---|---|---|---|---|---|---|
| Kajosaari L *et al.* (2004)[1] | 11 | - | - | - | 0.25 | - | - |
| Kajosaari L *et al.* (2005)[2] | 12 | - | - | - | 0.25 | - | - |
| Kalliokoski A *et al.* (2008) [3] | 13 | - | - | - | 0.5 | - | - |
| Kalliokoski A *et al.* (2008)[4] | 8 | - | - | - | 0.5 | - | - |
| Kalliokoski A *et al.* (2008)[5] | 16 | - | - | - | 0.25 | - | - |
| Tornio A *et al.* (2014)[6] | 8 | - | - | - | 0.25 | - | - |
| Kajosaari L *et al.* (2006)[7] | 10 | - | - | - | 0.25 | - | - |
| Kajosaari L *et al.* (2006)[8] | 10 | - | - | - | 0.25 | - | - |
| Niemi M *et al.* (2004)[9] | 6 | - | - | - | 0.25 | - | - |
| Niemi M *et al.* (2003)[10] | 12 | -[a] | 12 | - | 0.25 | 600 | 5 |
| Kalliokoski A *et al.* (2008)[11] | 22 | 22 | 22 | 22 | 0.25 | 600 | 5 |
| Tornio A *et al.* (2008)[12] | 10 | 10 | 10 | 10 | 0.25 | 600 | 5 |
| Backman J *et al.* (2009)[13] | 9 | 9 | 9 | 9 | 0.25 | 600 | 5 |
| Honkalammi J *et al.* (2011)[14] | 9 | 9 | 9 | 9 | 0.25 | 600 | 1 |
| Honkalammi J *et al.* (2011)[15] | 6 | 6 | 6 | 6 | 0.25 | 900 | 1 |
| Honkalammi J *et al.* (2012)[16] | 10 | 10 | 10 | 10 | 0.25 | 900 | 9 |
| Karonen T *et al.* (2012)[17] | - | - | 8 | 8 | - | 600 | 5 |
| Karonen T *et al.* (2010)[18] | - | - | 7 | 7 | - | 600 | 5 |
| Karonen T *et al.* (2011)[19] | - | - | 8 | 8 | - | 600 | 5 |
| Filppula A *et al.* (2013)[20] | - | - | 9 | 9 | - | 600 | 5 |
| Backman J *et al.* (2005)[21] | - | - | 9 | - | - | 600 | 5 |
| Tornio A *et al.* (2007)[22] | - | - | 7 | - | - | 600 | 5 |
| Niemi M *et al.* (2007)[23] | - | - | 7 | - | - | 600 | 5 |
| Niemi *et al.* (2003)[24] | - | - | 7 | - | - | 600 | 5 |
| unpublished | - | - | 9 | - | - | 600 | 5 |

| | | | | | | | |
|---|---|---|---|---|---|---|---|
| Tornio A *et al.* (2006)[25] | - | - | 9 | - | - | 600 | 5 |
| **Total** | **172** | **66** | **158** | **98** | | | |

[a] Only gemfibrozil-repaglinide interaction studies with gemfibrozil 1-O-β glucuronide concentration were included in the analyses with repaglinide after gemfibrozil pretreatment.

b.i.d., twice a day

**Table S2** Study settings of original pharmacokinetic studies.

| | Repaglinide | | | Gemfibrozil | | | Gemfibrozil 1-O-β glucuronide | |
|---|---|---|---|---|---|---|---|---|
| **Original study reference** | **LLQ (ng/mL)** | **CV** | **Sampling timepoints (h)** | **CV** | **LLQ (ng/mL)** | **Sampling timepoints (h)** | **LLQ (ng/mL)** | **CV** |
| Kajosaari L *et al.* (2004)[1] | 0.05 | ≤10 % | 0, 0.33, 0.67, 1, 1.33, 1.67, 2, 2.5, 3, 4, 5, 7 | | | | | |
| Kajosaari L *et al.* (2005)[2] | 0.02 | ≤14 % | 0, 0.33, 0.67, 1, 1.33, 1.67, 2, 2.5, 3, 4, 5, 7 | | | | | |
| Kalliokoski A *et al.* (2008) [3] | 0.01 | ≤10 % | 0, 0.25, 0.5, 0.75, 1, 1.25, 1.5, 1.75, 2, 2.5, 3, 4, 5, 7 | | | | | |
| Kalliokoski A *et al.* (2008)[4] | 0.05 | ≤5 % | 0, 0.25, 0.5, 0.75, 1, 1.25, 1.5, 1.75, 2, 2.5, 3, 4, 5, 7 | | | | | |
| Kalliokoski A *et al.* (2008)[5] | 0.05 | ≤9 % | 0, 0.25, 0.5, 0.75, 1, 1.25, 1.5, 1.75, 2, 2.5, 3, 4, 5, 7 | | | | | |
| Tornio A *et al.* (2014)[6] | 0.01 | ≤15 % | 0, 0.25, 0.5, 0.75, 1, 1.33, 1.67, 2, 2.5, 3, 4, 5, 7 | | | | | |
| Kajosaari L *et al.* (2006)[7] | 0.02 | ≤13 % | 0, 0.33, 0.67, 1, 1.33, 1.67, 2, 2.5, 3, 4, 5, 7 | | | | | |
| Kajosaari L *et al.* (2006)[8] | 0.01 | ≤9 % | 0, 0.33, 0.67, 1, 1.33, 1.67, 2, 2.5, 3, 4, 5, 7 | | | | | |
| Niemi M *et al.* (2004)[9] | 0.01 | ≤14 % | 0, 0.33, 0.67, 1, 1.33, 1.67, 2, 2.5, 3, 4, 5, 7 | | | | | |
| Niemi M *et al.* (2003)[10] | 0.1 | ≤13 % | 0, 0.33, 0.67, 1, 1.33, 1.67, 2, 2.5, 3, 4, 5, 7 | ≤11 % | 100 | 0, 0.75, 1.67, 2, 3, 4, 5, 6, 8 | | |
| Kalliokoski A *et al.* (2008)[11] | 0.01 | ≤8 % | 0, 0.25, 0.5, 0.75, 1, 1.25, 1.5, 1.75, 2, 2.5, 3, 4, 5, 7 | ≤12 % | 100 | 0, 1.25, 2, 4, 10 | 100 | ≤6 % |
| Tornio A *et al.* (2008)[12] | 0.02 | ≤6 % | 0, 0.33, 0.67, 1, 1.33, 1.67, 2, 2.5, 3, 4, 5, 7 | ≤9 % | 100 | 0, 0.33, 0.67, 1, 1.33, 1.67, 2, 2.5, 3, 4, 5, 7, 9 | 100 | ≤15 % |
| Backman J *et al.* (2009)[13] | 0.01 | ≤3 % | 0, 0.25, 0.33, 0.5, 0.67, 0.75, 1, 1.33, 1.67, 2, 2.5, 3, 4, 5, 7 | ≤4 % | 2.5 | 0, 0.92, 1.33, 1.67, 2, 2.33, 2.67, 3, 3.5, 4, 5, 6, 8, 10 | 2.5 | ≤8 % |
| Honkalammi J *et al.* (2011)[14] | 0.01 | ≤5 % | 0, 0.25, 0.5, 0.75, 1, 1.33, 1.67, 2, 2.5, 3, 4, 5, 7 | ≤4 % | 2.5 | 0.5, 0.92, 1.25, 1.5, 1.75, 2, 2.33, 2.67, 3, 3.5, 4, 5, 6, 8, 10 | 2.5 | ≤5 % |
| Honkalammi J *et al.* (2011)[15] | 0.01 | ≤5 % | 0, 0.25, 0.5, 0.75, 1, 1.33, 1.67, 2, 2.5, 3, 4, 5, 7 | ≤9 % | 2.5 | 0.5, 0.92, 1.25, 1.5, 1.75, 2, 2.33, 2.67, 3, 3.5, 4, 5, 6, 8, 10 | 2.5 | ≤7 % |

| | | | | | | | | |
|---|---|---|---|---|---|---|---|---|
| Honkalammi J *et al.* (2012)[16] | 0.01 | ≤10 % | 0, 0.25, 0.5, 0.75, 1, 1.33, 1.67, 2, 2.5, 3, 4, 5, 7 | ≤7 % | 2.5 | 0, 0.5, 0.92, 1.25, 1.5, 1.75, 2, 2.33, 2.67, 3, 3.5, 4, 5, 6, 8, 10 | 2.5 | ≤5 % |
| Karonen T *et al.* (2012)[17] | - | | | ≤6 % | 100 | 0, 2, 4, 6 | 50 | ≤10 % |
| Karonen T *et al.* (2010)[18] | - | | | ≤4 % | 5 | 0, 2, 4, 6, 10 | 5 | ≤8 % |
| Karonen T *et al.* (2011)[19] | - | | | ≤5 % | 5 | 0, 2, 4, 6, 10 | 5 | ≤8 % |
| Filppula A *et al.* (2013)[20] | - | | | ≤4 % | 250 | 0, 1.5, 2, 2.5, 3, 4, 5, 7, 9 | 250 | ≤7 % |
| Backman J *et al.* (2005)[21] | - | | | ≤4 % | 100 | 0, 1.5, 2, 2.5, 3, 3.5, 4, 5, 7, 9, 11 | | |
| Tornio A *et al.* (2007)[22] | - | | | ≤7 % | 100 | 0, 1.5, 2, 2.5, 3, 4, 5, 7, 9 | | |
| Niemi M *et al.* (2007)[23] | - | | | ≤7 % | 100 | 0, 2, 4, 6 | | |
| Niemi *et al.* (2003)[24] | - | | | ≤7 % | 100 | 0, 2, 3, 5, 8 | | |
| unpublished | - | | | ≤6 % | 100 | 0, 2, 3, 4, 5, 7, 9, 11 | | |
| Tornio A *et al.* (2006)[25] | - | | | ≤7 % | 100 | 0, 1.5, 2, 2.5, 3, 4, 5, 6, 7, 8, 10 | | |

CV, day-to-day coefficient of variation at relevant concentrations; LLQ, lower limit of quantification

**Table S3** Variants included in the candidate gene analysis of repaglinide.

| Gene | RsID | Nucleotide change | Amino acid change | Haplotype | MAF this study | MAF in 1000 genomes[26] | | | | |
|---|---|---|---|---|---|---|---|---|---|---|
| | | | | | | FIN | EUR[a] | EAS | SAS | AFR |
| *CYP2C8* | rs10509681 | c.1196A>G | p.Lys399Arg | *CYP2C8*3* | 0.064 | 0.081 | 0.118 | 0.001 | 0.030 | 0.008 |
| *CYP2C8* | rs1058930 | c.792C>G | p.Ile264Met | *CYP2C8*4* | 0.055 | 0.081 | 0.058 | 0 | 0.007 | 0.004 |
| *CYP2C8* | rs2071426 | c.168+669A>G | - | *CYP2C8*19* | 0.317 | 0.268 | 0.283 | 0.059 | 0.220 | 0.256 |
| *SLCO1B1* | rs2306283 | c.388A>G | p.Asn130Asp | *SLCO1B1*14, *15, *20, *37* | 0.471 | 0.439 | 0.403 | 0.762 | 0.547 | 0.818 |
| *SLCO1B1* | rs11045819 | c.463C>A | p.Pro155Thr | *SLCO1B1*14* | 0.113 | 0.076 | 0.144 | 0.003 | 0.030 | 0.060 |
| *SLCO1B1* | rs4149056 | c.521T>C | p.Val174Ala | *SLCO1B1*5, *15* | 0.250 | 0.182 | 0.161 | 0.123 | 0.043 | 0.014 |
| *SLCO1B1* | rs34671512 | c.1929A>C | p.Leu643Phe | *SLCO1B1*20* | 0.049 | 0.040 | 0.052 | 0.005 | 0.049 | 0.065 |
| *SLCO1C1* | rs10841611 | c.1947T>C | p.His649His | - | 0.477 | 0.505 | 0.504 | 0.591 | 0.646 | 0.195 |

MAF of the candidate gene variants is shown in 172 participants with repaglinide and in 1000 genomes Project Phase 3. In some of the original repaglinide studies participants were recruited based on *SLCO1B1* genotype, and therefore, allele frequencies may deviate from the general Finnish population.

AFR, African; EUR, European; EAS, East Asian; FIN, Finnish in Finland; MAF, minor allele frequency; SAS, South Asian

[a]European population includes also Finnish individuals

**Table S4** Results of the candidate gene analysis for montelukast $AUC_{0-\infty}$ in 191 healthy participants.

| Variable | MAF | Effect size [a] (90% CI) | *P* value | Adjusted $R^2$ |
|---|---|---|---|---|
| BSA | - | -11.0% (-8,6%; -13.4%) | $2.02 \times 10^{-8}$ | 0.16 |
| *UGT1A3*2* | 0.39 | -16.7% (-21.0%; -12.3%) | $4.06 \times 10^{-8}$ | 0.31 |
| *ABCC9* rs704212 | 0.14 | -14.3% (-20.4%; -7.7%) | $7.32 \times 10^{-4}$ | 0.36 |
| *CYP2C8*19* (rs2071426) | 0.27 | 13.1% (7.0%; 19.5%) | $3.67 \times 10^{-4}$ | 0.41 |
| *CYP2C8*4* (rs1058930) | 0.07 | 17.4% (6.8%; 29.2%) | $6.01 \times 10^{-3}$ | 0.44 |

$AUC_{0-\infty}$, area under the plasma concentration-time curve from 0 h to infinity; BSA, Body surface area; CI, confidence interval; MAF, minor allele frequency.

[a]BSA effect per 10% increase; genetic variant effect per copy of the minor allele

**Table S5** Pharmacokinetic variables of repaglinide and gemfibrozil, and their associations with covariates.

| Variable | *n* | Geometric mean | Geo CV | Range | Covariate | Covariate effect (90% CI) | *P*-value |
|---|---|---|---|---|---|---|---|
| Repaglinide $AUC_{0-\infty}$ no concomitant medication (ng×h/mL) | 172 | 4.6 | 47.6% | 1.1-18.1 | Weight[a] | -7% (-10%, -3%) | 0.0032 |
| | | | | | Sex[b] | 25% (9%, 43%) | 0.0079 |
| Repaglinide $AUC_{0-\infty}$ after gemfibrozil pretreatment (ng×h/mL) | 66 | 35.6 | 30.3% | 18.7-68.3 | Gemfibrozil glucuronide[c] | 23% (13%, 35%) | $2.6 \times 10^{-4}$ |
| | | | | | Sex[b] | 18% (5%, 34%) | 0.024 |
| Fold increase in repaglinide $AUC_{0-\infty}$ caused by gemfibrozil | 66 | 7.6 | 35.0% | 2.9-16.9 | Weight[a] | 6% (2%, 12%) | 0.029 |
| Gemfibrozil AUC (ng×h/mL) | 158 | 90.3 | 32.5% | 44.0-204.5 | Weight[a] | -9% (-11%, -7%) | $8.2 \times 10^{-12}$ |
| Gemfibrozil 1-O-β-glucuronide AUC (ng×h/mL) | 98 | 100.9 | 35.1% | 36.8-215.5 | Number of gemfibrozil doses[d] | -35% (-43%, -26%) | $2.5 \times 10^{-7}$ |
| | | | | | Weight[a] | -8% (-11%, -6%) | $9.0 \times 10^{-6}$ |
| Gemfibrozil 1-O-β-glucuronide / gemfibrozil AUC ratio | 98 | 1.1 | 31.2% | 0.7-2.9 | Number of gemfibrozil doses[d] | -25% (-35%, -15%) | $4.6 \times 10^{-4}$ |

[a]Effect per 10% increase in body weight

[b]Effect women vs. men

[c]Effect per 100% increased in gemfibrozil 1-O-β-glucuronide $AUC_{0-12h}$

[d]Number of gemfibrozil doses single vs. multiple doses

$AUC_{0-\infty}$, area under the plasma concentration-time curve from 0 hours to infinity; CI, confidence interval; Geo CV, geometric coefficient of variation

**Table S6** The associations of *SLCO1C1* rs10841611 with the OATP1B1 biomarker GCDCA-3G and the OATP1B3 biomarker GCDCA-S in 356 healthy participants.

| **Trait** | **Genotype (*n*)** | **Geometric mean ng/mL (90% CI)** | **GMR (90% CI)** | ***P*-value** |
|---|---|---|---|---|
| GCDCA-3G | | | | |
| | rs10841611 T>C | | | |
| | TT (106) | 40.7 (32.6, 50.6) | 1 | |
| | TC (167) | 39.8 (32.4, 48.9) | 0.98 (0.72, 1.32) | 0.90 |
| | CC (83) | 42.7 (33, 55.2) | 1.05 (0.75, 1.47) | 0.81 |
| GCDCA-3S | | | | |
| | rs10841611 T>C | | | |
| | TT (106) | 69.5 (62.5, 77.4) | 1 | |
| | TC (167) | 60.3 (55.4, 65.6) | 0.87 (0.76, 0.99) | 0.086 |
| | CC (83) | 61 (54.1, 68.8) | 0.88 (0.75, 1.03) | 0.18 |

GCDCA-3G data were adjusted for sex and *SLCO1B1* genotype, and GCDCA-3S data for sex.

CI, confidence interval; GCDCA-3G, glycochenodeoxycholate 3-O-glucuronide; GCDCA-3S, glycochenodeoxycholic acid 3-O-sulfate; GMR geometric mean ratio.

**Table S7** The associations of *SLCO1C1* rs10841611 with the AUC of gemfibrozil and gemfibrozil 1-O-β glucuronide.

| Trait | Genotype (*n*) | Geomean (90% CI) | GMR (90% CI) | *P*-value |
|---|---|---|---|---|
| | rs10841611 T>C | | | |
| Gemfibrozil AUC | | | | |
| | TT (38) | 93.7 (86.1, 102.1) | 1 | |
| | TC (78) | 89.5 (84.4, 95.0) | 0.96 (0.86, 1.06) | 0.47 |
| | CC (42) | 88.7 (81.8, 96.2) | 0.95 (0.84, 1.06) | 0.44 |
| Gemfibrozil 1-O-β glucuronide AUC | | | | |
| | TT (26) | 102.3 (91.5, 114.4) | 1 | |
| | TC (47) | 99.1 (91.2, 107.8) | 0.97 (0.84, 1.11) | 0.71 |
| | CC (25) | 102.7 (91.6, 115.1) | 1.00 (0.86, 1.18) | 0.97 |

AUC, area under the plasma concentration-time curve; CI, confidence interval; GMR geometric mean ratio.

**Table S8** Frequencies of *CYP2C8* core alleles and the alleles containing rs2071426 in continental superpopulations in the 1000 genomes data.[26]

| *CYP2C8* allele | Defining variants | African | American | East Asian | European | South Asian |
|---|---|---|---|---|---|---|
| *CYP2C8*1* | - | 72.2% | 72.6% | 93.1% | 53.0% | 73.8% |
| *CYP2C8*2* | c.805A>T (rs11572103, p.I269F), c.168+669A>G (rs2071426) | 18.8% | 1.0% | 0.0% | 0.4% | 1.1% |
| *CYP2C8*3* | c.416G>A (rs11572080, p.R139K), c.1196A>G (rs10509681, p.K399R) | 0.8% | 9.9% | 0.1% | 11.8% | 3.0% |
| *CYP2C8*4* | c.792C>G (rs1058930, p.I264M) | 0.4% | 1.9% | 0.0% | 5.8% | 0.7% |
| *CYP2C8*6* | c.511G>A (rs142886225, p.G171S) | 0.0% | 0.0% | 0.6% | 0.0% | 0.0% |
| *CYP2C8*11* | c.820G>T (rs78637571, p.E274X) | 0.0% | 0.0% | 0.3% | 0.0% | 0.0% |
| *CYP2C8*14* | c.712G>C (rs188934928, A238P) | 0.0% | 0.0% | 0.1% | 0.0% | 0.0% |
| *CYP2C8*15* | c.541G>A (rs41286886, p.V181I) | 0.0% | 0.3% | 0.0% | 1.1% | 0.0% |
| *CYP2C8*16* | c.992T>C (rs146806199, p.I331T) | 0.2% | 0.0% | 0.0% | 0.0% | 0.4% |
| *CYP2C8*17* | c.730A>G (rs11572102, p.I244V) | 0.7% | 0.0% | 0.0% | 0.0% | 0.0% |
| *CYP2C8*19* | c.168+669A>G (rs2071426) | 6.8% | 14.2% | 5.9% | 27.9% | 20.9% |
| *CYP2C8*20* | c.805A>T (rs11572103, p.I269F) | 0.2% | 0.1% | 0.0% | 0.0% | 0.1% |

## *CYP2C8*

| allele | *n* | frequency | c.*24C>T rs1058932 | c.1196A>G, p.Lys399Arg rs10509681 | c.1149+49T>A rs2275620 | c.792C>G, p.Ile264Met rs1058930 | c.482-21T>A rs7098376 | c.481+43G>C rs11572082 | c.416G>A, p.Arg139Lys rs11572080 | c.332-4T>C rs11572079 | c.168+669A>G rs2071426 | g.-271C>A rs7909236 | g.-370T>G rs17110453 | g.-411C>T rs7912549 |
|---|---|---|---|---|---|---|---|---|---|---|---|---|---|---|
| *1 | 98 | 24.5% | C | A | T | C | T | G | G | T | A | A | T | T |
| *1 | 52 | 13.0% | C | A | T | C | T | G | G | T | A | C | T | C |
| *1 | 43 | 10.8% | T | A | A | C | T | G | G | T | A | C | G | T |
| *1 | 12 | 3.0% | T | A | A | C | T | G | G | T | A | C | T | T |
| *1 | 10 | 2.5% | T | A | A | C | T | G | G | C | A | C | G | T |
| *1 | 4 | 1.0% | C | A | A | C | T | G | G | T | A | C | T | T |
| *3 | 41 | 10.3% | C | G | A | C | T | C | A | T | A | C | T | T |
| *4 | 19 | 4.8% | T | A | A | G | T | G | G | T | A | C | G | T |
| *19 | 112 | 28.0% | C | A | A | C | A | G | G | T | G | C | T | T |

**Figure S1** *CYP2C8* haplotypes in 200 healthy unrelated Finnish participants from a previous pharmacogenetic study.[27] Haplotypes with less than three observations were excluded from the figure. Missense variants are marked with dark green, a splice site variant with yellow, and other variants with light green color.

SLCO1C1 | SLCO1B3 | SLCO1B3-SLCO1B7 | SLCO1B1 | SLCO1A2

| | rs972505 | rs10841611 | rs6487138 (*SLCO1C1* p.Ser677Phe) | rs953001 | rs953002 | rs10444412 | rs1304539 | rs4149117 (*SLCO1B3* p.Ala112Ser) | rs7311358 (*SLCO1B3* p.Ile233Met) | rs10841684 | rs11045676 (*SLCO1B3-SLCO1B7* p.Phe295Tyr) | rs11045681 (*SLCO1B3-SLCO1B7* p.Tyr358Cys) | rs11045797 | rs2306283 (*SLCO1B1* p.Asn130Asp) | rs11045819 (*SLCO1B1* p.Pro155Thr) | rs4149056 (*SLCO1B1* p.Val174Ala) | rs11045863 | rs4363657 | rs34671512 (*SLCO1B1* p.Leu643Phe) | rs11045891 | rs11045906 | rs71446763 | rs10841781 | rs11045916 | rs11045917 | rs11045918 | rs4149006 | rs6487215 | rs10841795 (*SLCO1A2* Ile13Thr) |
|---|---|---|---|---|---|---|---|---|---|---|---|---|---|---|---|---|---|---|---|---|---|---|---|---|---|---|---|---|---|
| rs972505 | - | 1 | 0.95 | 0.95 | 0.95 | 0.95 | 0.04 | 0.05 | 0.05 | 0.05 | 0.05 | 0.05 | 0.05 | 0.00 | 0.03 | 0.09 | 0.04 | 0.03 | 0.05 | 0.06 | 0.06 | 0.06 | 0.10 | 0.06 | 0.06 | 0.06 | 0.11 | 0.06 | 0.06 |
| rs10841611 | 1 | - | 0.95 | 0.95 | 0.95 | 0.95 | 0.04 | 0.05 | 0.05 | 0.05 | 0.05 | 0.05 | 0.05 | 0.00 | 0.03 | 0.09 | 0.04 | 0.03 | 0.05 | 0.06 | 0.06 | 0.06 | 0.10 | 0.06 | 0.06 | 0.06 | 0.11 | 0.06 | 0.06 |
| rs6487138 (*SLCO1C1* p.Ser677Phe) | 0.98 | 0.98 | - | 1 | 1 | 1 | 0.03 | 0.04 | 0.04 | 0.04 | 0.05 | 0.05 | 0.05 | 0.00 | 0.03 | 0.08 | 0.04 | 0.02 | 0.02 | 0.06 | 0.05 | 0.06 | 0.10 | 0.06 | 0.06 | 0.06 | 0.11 | 0.06 | 0.06 |
| rs953001 | 0.98 | 0.98 | 1 | - | 1 | 1 | 0.03 | 0.04 | 0.04 | 0.04 | 0.05 | 0.05 | 0.05 | 0.00 | 0.03 | 0.08 | 0.04 | 0.02 | 0.02 | 0.06 | 0.05 | 0.06 | 0.10 | 0.06 | 0.06 | 0.06 | 0.11 | 0.06 | 0.06 |
| rs953002 | 0.98 | 0.98 | 1 | 1 | - | 1 | 0.03 | 0.04 | 0.04 | 0.04 | 0.05 | 0.05 | 0.05 | 0.00 | 0.03 | 0.08 | 0.04 | 0.02 | 0.02 | 0.06 | 0.05 | 0.06 | 0.10 | 0.06 | 0.06 | 0.06 | 0.11 | 0.06 | 0.06 |
| rs10444412 | 0.98 | 0.98 | 1 | 1 | 1 | - | 0.03 | 0.04 | 0.04 | 0.04 | 0.05 | 0.05 | 0.05 | 0.00 | 0.03 | 0.08 | 0.04 | 0.02 | 0.02 | 0.06 | 0.05 | 0.06 | 0.10 | 0.06 | 0.06 | 0.06 | 0.11 | 0.06 | 0.06 |
| rs1304539 | 0.36 | 0.36 | 0.32 | 0.32 | 0.32 | 0.32 | - | 0.99 | 0.99 | 0.99 | 0.05 | 0.05 | 0.05 | 0.06 | 0.05 | 0.37 | 0.05 | 0.20 | 0.02 | 0.05 | 0.07 | 0.03 | 0.06 | 0.03 | 0.03 | 0.03 | 0.05 | 0.03 | 0.03 |
| rs4149117 (*SLCO1B3* p.Ala112Ser) | 0.37 | 0.37 | 0.33 | 0.33 | 0.33 | 0.33 | 1 | - | 1 | 1 | 0.05 | 0.05 | 0.05 | 0.05 | 0.05 | 0.36 | 0.05 | 0.19 | 0.02 | 0.05 | 0.07 | 0.03 | 0.06 | 0.03 | 0.03 | 0.03 | 0.06 | 0.03 | 0.03 |
| rs7311358 (*SLCO1B3* p.Ile233Met) | 0.37 | 0.37 | 0.33 | 0.33 | 0.33 | 0.33 | 1 | 1 | - | 1 | 0.05 | 0.05 | 0.05 | 0.05 | 0.05 | 0.36 | 0.05 | 0.19 | 0.02 | 0.05 | 0.07 | 0.03 | 0.06 | 0.03 | 0.03 | 0.03 | 0.06 | 0.03 | 0.03 |
| rs10841684 | 0.37 | 0.37 | 0.33 | 0.33 | 0.33 | 0.33 | 1 | 1 | 1 | - | 0.05 | 0.05 | 0.05 | 0.05 | 0.05 | 0.36 | 0.05 | 0.19 | 0.02 | 0.05 | 0.07 | 0.03 | 0.06 | 0.03 | 0.03 | 0.03 | 0.06 | 0.03 | 0.03 |
| rs11045676 (*SLCO1B3-SLCO1B7* p.Phe295Tyr) | 0.57 | 0.57 | 0.57 | 0.57 | 0.57 | 0.57 | 1 | 1 | 1 | 1 | - | 1 | 0.92 | 0.04 | 0.66 | 0.04 | 0.55 | 0.07 | 0.00 | 0.58 | 0.43 | 0.44 | 0.23 | 0.43 | 0.44 | 0.44 | 0.23 | 0.44 | 0.44 |
| rs11045681 (*SLCO1B3-SLCO1B7* p.Tyr358Cys) | 0.56 | 0.56 | 0.56 | 0.56 | 0.56 | 0.56 | 1 | 1 | 1 | 1 | 1 | - | 0.92 | 0.05 | 0.66 | 0.04 | 0.55 | 0.07 | 0.00 | 0.58 | 0.43 | 0.44 | 0.23 | 0.43 | 0.44 | 0.44 | 0.23 | 0.44 | 0.44 |
| rs11045797 | 0.57 | 0.57 | 0.57 | 0.57 | 0.57 | 0.57 | 1 | 1 | 1 | 1 | 0.97 | 0.97 | - | 0.07 | 0.7 | 0.04 | 0.58 | 0.06 | 0.00 | 0.62 | 0.46 | 0.47 | 0.26 | 0.46 | 0.47 | 0.47 | 0.26 | 0.47 | 0.47 |
| rs2306283 (*SLCO1B1* p.Asn130Asp) | 0.05 | 0.05 | 0.03 | 0.03 | 0.03 | 0.03 | 0.38 | 0.36 | 0.36 | 0.36 | 0.55 | 0.55 | 0.69 | - | 0.14 | 0.24 | 0.17 | 0.29 | 0.06 | 0.16 | 0.10 | 0.13 | 0.20 | 0.13 | 0.13 | 0.13 | 0.21 | 0.13 | 0.13 |
| rs11045819 (*SLCO1B1* p.Pro155Thr) | 0.48 | 0.48 | 0.48 | 0.48 | 0.48 | 0.48 | 1 | 1 | 1 | 1 | 0.82 | 0.82 | 0.85 | 1 | - | 0.04 | 0.85 | 0.06 | 0.00 | 0.84 | 0.64 | 0.64 | 0.37 | 0.64 | 0.64 | 0.64 | 0.37 | 0.64 | 0.64 |
| rs4149056 (*SLCO1B1* p.Val174Ala) | 0.55 | 0.55 | 0.50 | 0.50 | 0.50 | 0.50 | 0.63 | 0.63 | 0.63 | 0.63 | 1 | 1 | 1 | 0.81 | 1 | - | 0.05 | 0.67 | 0.02 | 0.05 | 0.06 | 0.03 | 0.05 | 0.03 | 0.03 | 0.03 | 0.05 | 0.03 | 0.03 |
| rs11045863 | 0.48 | 0.48 | 0.48 | 0.48 | 0.48 | 0.48 | 1 | 1 | 1 | 1 | 0.79 | 0.79 | 0.82 | 1 | 1 | 1 | - | 0.07 | 0.01 | 0.95 | 0.72 | 0.73 | 0.41 | 0.72 | 0.73 | 0.73 | 0.41 | 0.73 | 0.73 |
| rs4363657 | 0.24 | 0.24 | 0.20 | 0.20 | 0.20 | 0.20 | 0.53 | 0.51 | 0.51 | 0.51 | 1 | 1 | 1 | 0.73 | 1 | 1 | 1 | - | 0.03 | 0.07 | 0.09 | 0.04 | 0.00 | 0.04 | 0.04 | 0.04 | 0.00 | 0.04 | 0.04 |
| rs34671512 (*SLCO1B1* p.Leu643Phe) | 1 | 1 | 0.69 | 0.69 | 0.69 | 0.69 | 1 | 1 | 1 | 1 | 0 | 0 | 0.01 | 1 | 0.06 | 1 | 0.12 | 1 | - | 0.00 | 0.00 | 0.00 | 0.00 | 0.00 | 0.00 | 0.00 | 0.00 | 0.00 | 0.00 |
| rs11045891 | 0.60 | 0.60 | 0.60 | 0.60 | 0.60 | 0.60 | 1 | 1 | 1 | 1 | 0.79 | 0.79 | 0.82 | 1 | 0.97 | 1 | 1 | 1 | 0.06 | - | 0.77 | 0.77 | 0.44 | 0.76 | 0.77 | 0.77 | 0.44 | 0.77 | 0.77 |
| rs11045906 | 0.53 | 0.53 | 0.48 | 0.48 | 0.48 | 0.48 | 1 | 1 | 1 | 1 | 0.78 | 0.78 | 0.81 | 0.70 | 0.97 | 1 | 0.95 | 1 | 0.17 | 1 | - | 0.58 | 0.4 | 0.58 | 0.58 | 0.58 | 0.36 | 0.58 | 0.58 |
| rs71446763 | 0.67 | 0.67 | 0.67 | 0.67 | 0.67 | 0.67 | 0.84 | 0.85 | 0.85 | 0.85 | 0.70 | 0.71 | 0.73 | 1 | 0.84 | 0.83 | 0.97 | 0.84 | 0.04 | 0.97 | 0.97 | - | 0.60 | 1 | 1 | 1 | 0.61 | 1 | 1 |
| rs10841781 | 0.68 | 0.68 | 0.68 | 0.68 | 0.68 | 0.68 | 0.90 | 0.90 | 0.90 | 0.90 | 0.59 | 0.60 | 0.62 | 0.95 | 0.76 | 0.89 | 0.74 | 0.01 | 0.28 | 0.79 | 0.66 | 1 | - | 0.59 | 0.60 | 0.60 | 0.98 | 0.60 | 0.61 |
| rs11045916 | 0.67 | 0.67 | 0.67 | 0.67 | 0.67 | 0.67 | 0.84 | 0.84 | 0.84 | 0.84 | 0.70 | 0.70 | 0.72 | 1 | 0.84 | 0.82 | 0.97 | 0.84 | 0.04 | 0.97 | 0.96 | 1 | 1 | - | 1 | 1 | 0.61 | 1 | 1 |
| rs11045917 | 0.67 | 0.67 | 0.67 | 0.67 | 0.67 | 0.67 | 0.84 | 0.85 | 0.85 | 0.85 | 0.70 | 0.71 | 0.73 | 1 | 0.84 | 0.83 | 0.97 | 0.84 | 0.04 | 0.97 | 0.97 | 1 | 1 | 1 | - | 1 | 0.61 | 1 | 1 |
| rs11045918 | 0.67 | 0.67 | 0.67 | 0.67 | 0.67 | 0.67 | 0.84 | 0.85 | 0.85 | 0.85 | 0.70 | 0.71 | 0.73 | 1 | 0.84 | 0.83 | 0.97 | 0.84 | 0.04 | 0.97 | 0.97 | 1 | 1 | 1 | 1 | - | 0.61 | 1 | 1 |
| rs4149006 | 0.72 | 0.72 | 0.72 | 0.72 | 0.72 | 0.72 | 0.90 | 0.90 | 0.90 | 0.90 | 0.58 | 0.58 | 0.60 | 1 | 0.74 | 0.89 | 0.72 | 0.01 | 0.22 | 0.77 | 0.60 | 1 | 1 | 1 | 1 | 1 | - | 0.61 | 0.63 |
| rs6487215 | 0.67 | 0.67 | 0.67 | 0.67 | 0.67 | 0.67 | 0.84 | 0.85 | 0.85 | 0.85 | 0.70 | 0.71 | 0.73 | 1 | 0.84 | 0.83 | 0.97 | 0.84 | 0.04 | 0.97 | 0.97 | 1 | 1 | 1 | 1 | 1 | 1 | - | 1 |
| rs10841795 (*SLCO1A2* Ile13Thr) | 0.68 | 0.68 | 0.68 | 0.68 | 0.68 | 0.68 | 0.84 | 0.85 | 0.85 | 0.85 | 0.70 | 0.70 | 0.73 | 1 | 0.84 | 0.83 | 0.97 | 0.84 | 0.03 | 0.97 | 0.97 | 1 | 1 | 1 | 1 | 1 | 1 | 1 | - |

$r^2$: 1.00 | 0.90-0.99 | 0.70-0.89 | 0.50-0.69 | 0.30-49 | 0.10-29

*D′*

**Figure S2** Linkage disequilibrium of the genome-wide significant variants from the GWAS and *SLCO1B1* missense variants.

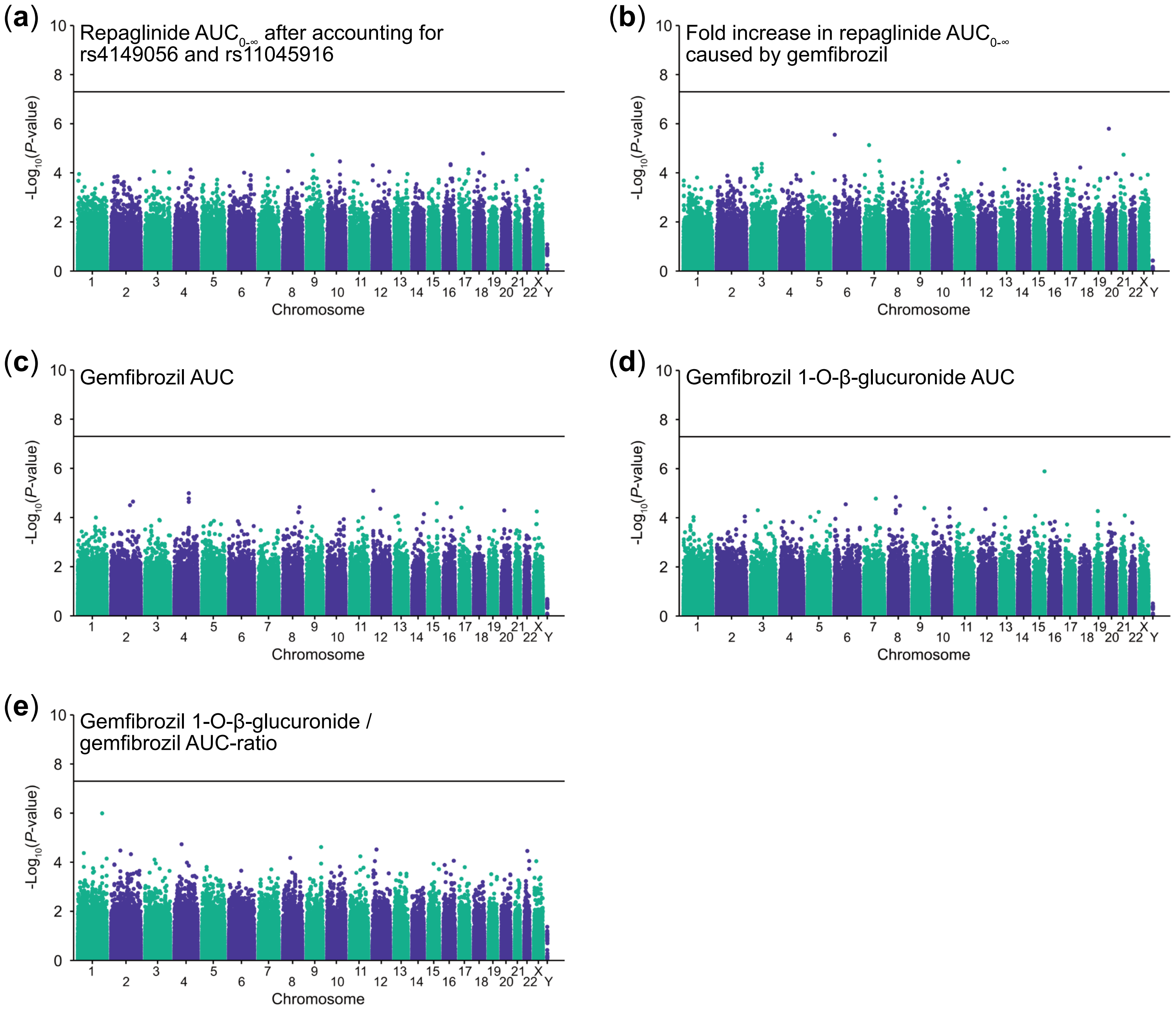


**Figure S3** Manhattan plots of (a) repaglinide $AUC_{0-\infty}$ in 172 participants after accounting for rs4149056 and rs11045916, (b) fold increase in repaglinide $AUC_{0-\infty}$ caused by gemfibrozil in 66 participants, (c) gemfibrozil AUC in 158 participants, (d) gemfibrozil 1-O-β-glucuronide AUC, and (e) gemfibrozil 1-O-β-glucuronide / gemfibrozil AUC-ratio in 98 participants. Horizontal lines indicate the genome-wide significance level of $5\times10^{-8}$

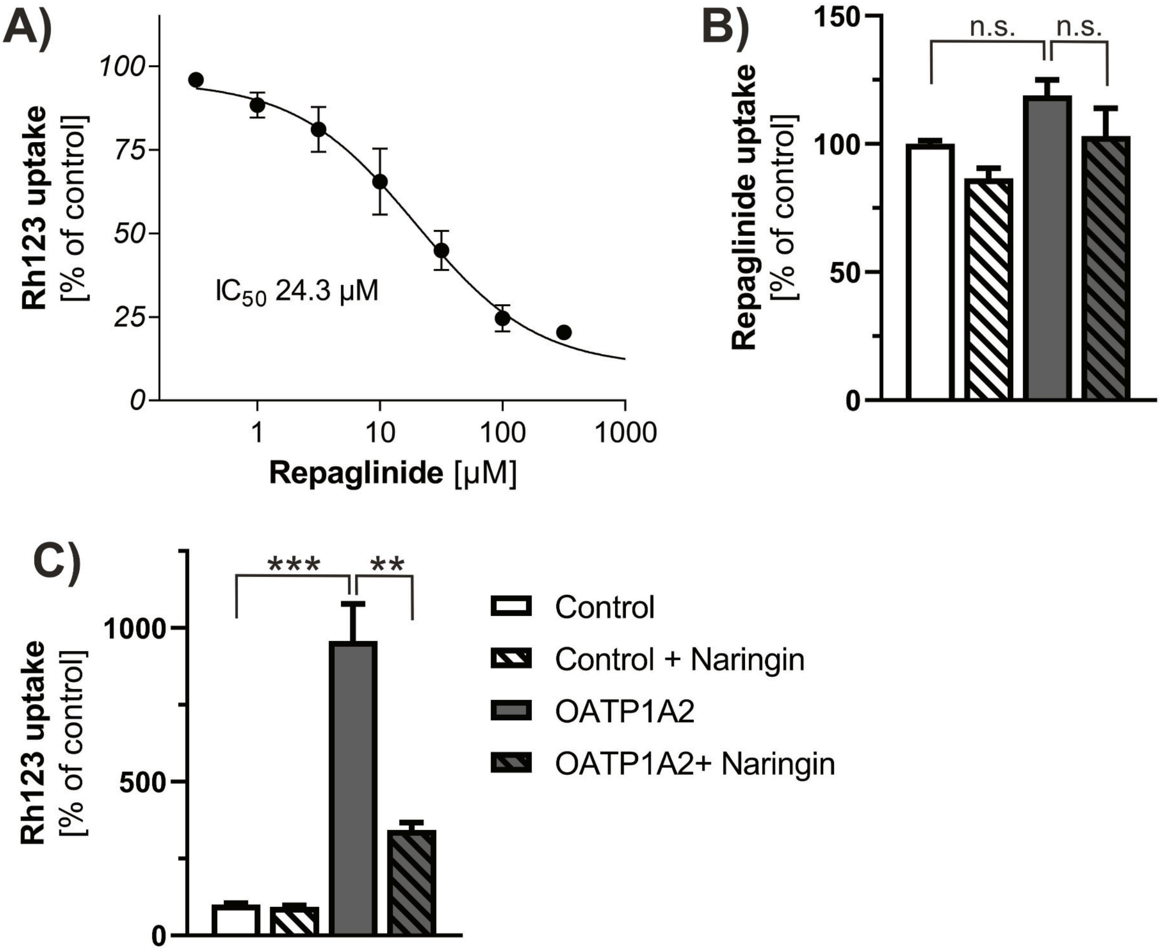


**Figure S4** In vitro analysis of the interaction between repaglinide and OATP1A2. We utilized MDCKII cells genetically engineered to overexpress human OATP1A2 to evaluate (A) the potential of repaglinide to inhibit the uptake of the model OATP1A2 substrate rhodamine123 (Rh123, 1 µM) and (B) direct uptake of repaglinide via OATP1A2 (100 nM, 5 min incubation time, *n* = 3). To selectively inhibit OATP1A2-mediated uptake, 500 µM naringin was used. As a positive control, the uptake of rhodamine 123 was measured under the same conditions as repaglinide (C). Significance was calculated using ANOVA with Tukey post-hoc test. *** $p<0.001$, ** $p<0.01$, n.s. not significant.

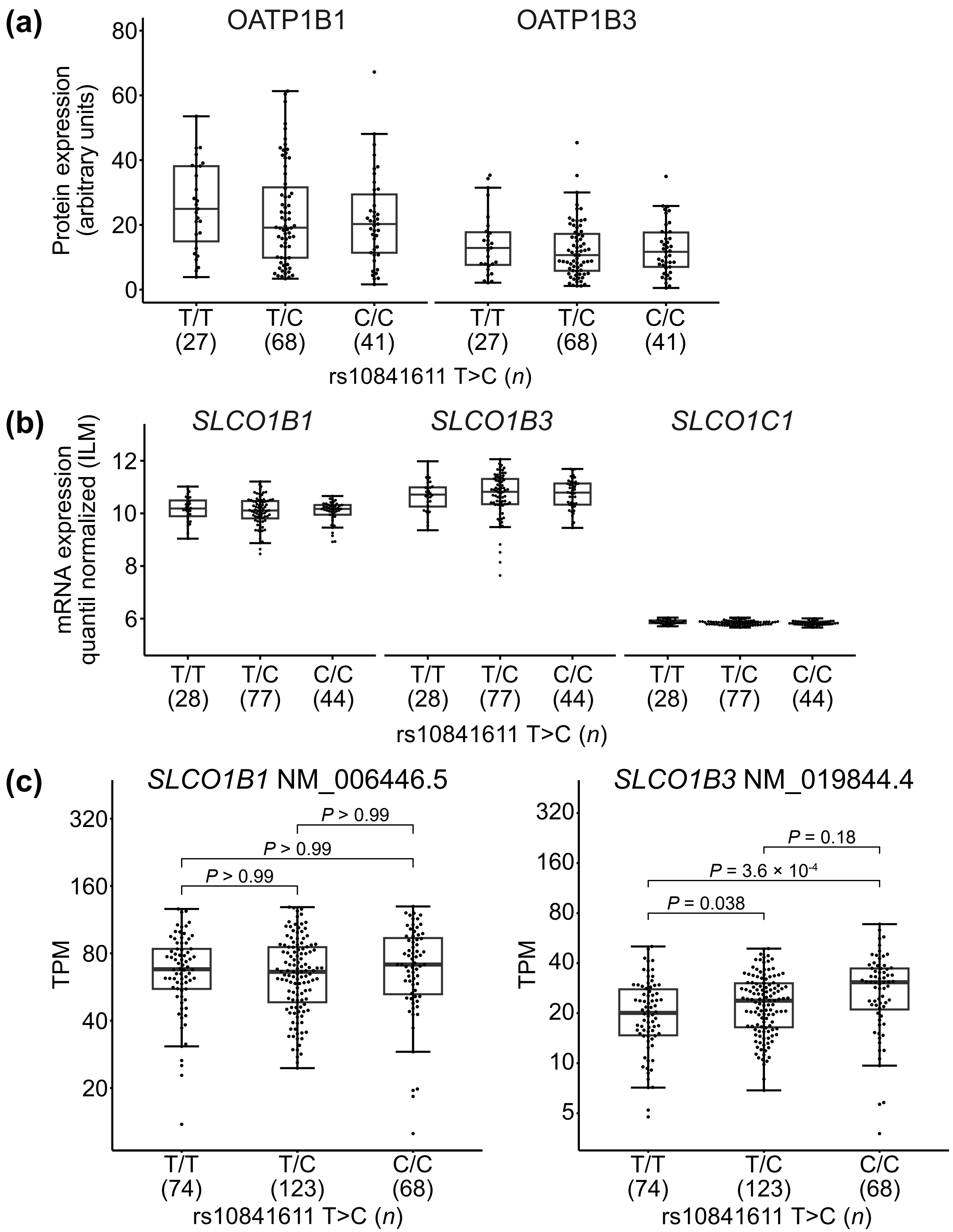


**Figure S5** Associations of *SLCO1C1* rs10841611 T>C in human liver samples with: (**a**) OATP1B1 and OATP1B3 protein expression; (**d**) *SLCO1B1*, *SLCO1B3*, and *SLCO1C1* total mRNA expression; (**c**) *SLCO1B1* and *SLCO1B3* transcript expression ($n$ = 265). In (**a**) and (**b**) no statistically significant differences were observed between groups. In (**c**) expression of *SLCO1C1* transcripts was low and below filter thresholds

TPM, trancsripts per million

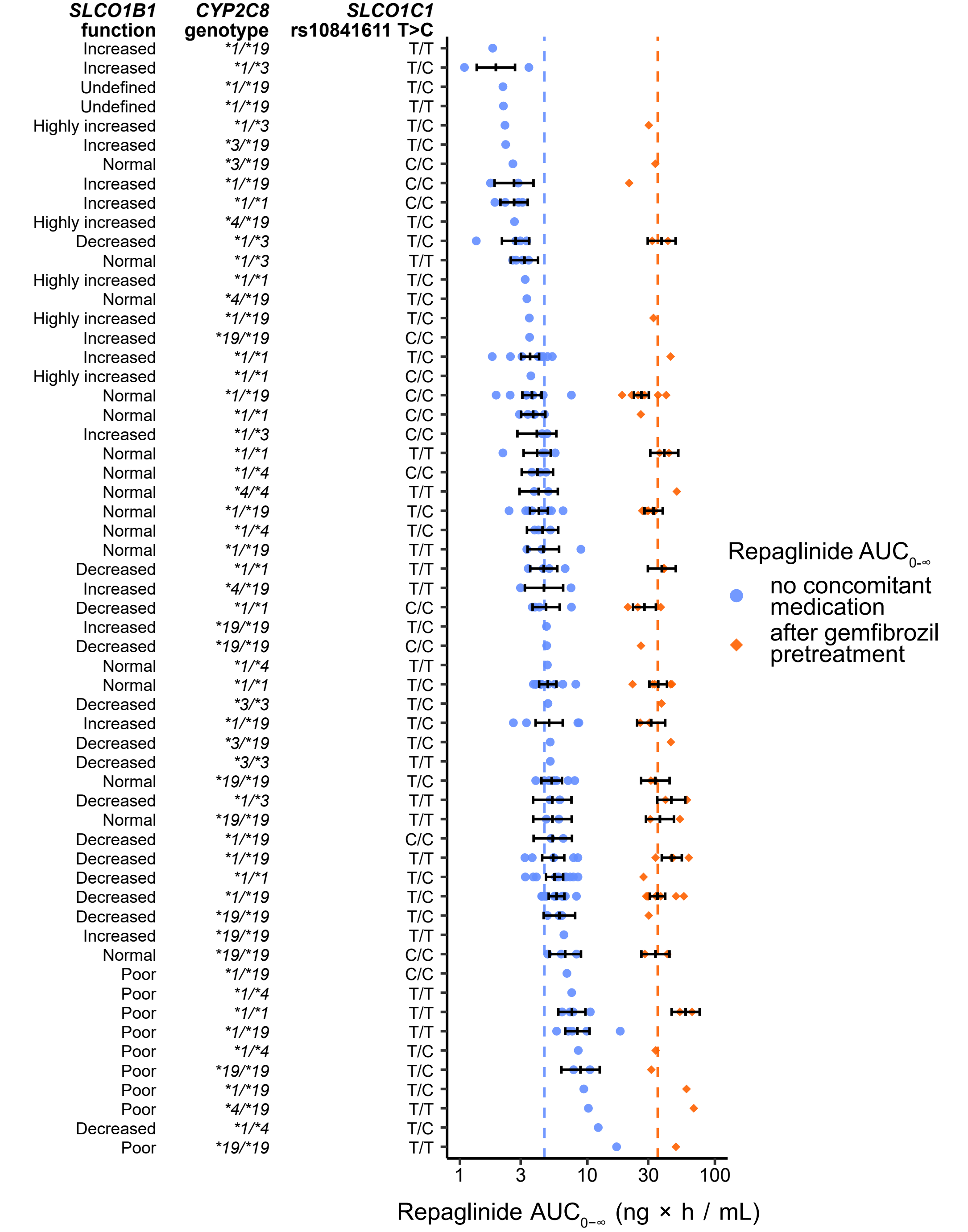


**Figure S6** The $AUC_{0\text{-}\infty}$ values of repaglinide by different *SLCO1B1*, *CYP2C8*, and *SLCO1C1* genotype groups. Blue circles represent individual $AUC_{0\text{-}\infty}$ values of repaglinide without concomitant medication and orange diamonds represent $AUC_{0\text{-}\infty}$ values of repaglinide after gemfibrozil pretreatment. Vertical lines with whiskers indicate geometric means with 90% confidence intervals. Dashed vertical lines indicate geometric mean $AUC_{0\text{-}\infty}$ of repaglinide: blue without concomitant medications ($n$ = 172) and orange after gemfibrozil pretreatment ($n$ = 66).

| | | | | *SLCO1B1* | | | | | | | | | | | | | | | | | *SLCO1A2* | | | | | | | | | | | | | | | | | | | | | | | | | | | | | | | | |
|---|---|---|---|---|---|---|---|---|---|---|---|---|---|---|---|---|---|---|---|---|---|---|---|---|---|---|---|---|---|---|---|---|---|---|---|---|---|---|---|---|---|---|---|---|---|---|---|---|---|---|---|---|---|
| *SLCO1B1* allele | CPIC clinical function | *n* | frequency | g.-10499A>C rs59710386 | c.-61-21A>C rs750325186 | c.388A>G, p.Asn130Asp rs2306283 | c.411G>A rs11045818 | c.463C>A, p.Pro155Thr rs11045819 | c.521T>C, p.Val174Ala rs4149056 | c.571T>C rs4149057 | c.597C>T rs2291075 | c.727+33C>T rs2291076 | c.1747+9A>G rs71577817 | c.1929A>C, p.Leu643Phe rs34671512 | c.*167T>C rs4149085 | c.*439T>G rs4149087 | c.*449A>C rs11045891 | c.*463A>G rs4149088 | c.*575G>A rs61760249 | rs10841777 | c.*4583A>C rs10841778 | c.*4574A>G rs10841779 | c.*4499G>C rs12581327 | c.*4431A>G rs875234 | c.*4339G>A rs10841780 | c.*4084C>T rs71446763 | c.*3117G>A rs61926244 | c.*3087T>C rs10841781 | c.*3042T>A rs11045916 | c.*2685C>T rs117186831 | c.*2576C>T rs116992851 | c.*2534C>T rs11045917 | c.*2011G>A rs4149009 | c.*1967T>C rs543717663 | c.*1770G>T rs11045918 | c.*1443C>T rs4149008 | c.*1227G>A rs4149007 | c.*1139A>G rs12317843 | c.*712C>A rs4149006 | c.*443C>T rs140638980 | c.*417G>A rs41275202 | c.*229A>C rs11045919 | c.*117C>A rs4140389 | c.1662C>T rs11568565 | c.1611-13A>G rs3764044 | c.1272-22G>A rs722994 | c.516A>C, p.Glu172Asp rs11568563 | c.61-34A>T rs11568570 | c.38T>C, p.Ile13Thr rs10841795 | c.-196C>G rs78221858 | c.-225C>T rs10841803 | c.-425T>C rs10841804 | c.-500A>G rs11046013 |
| *1 | Normal | 74 | 18.5% | A | A | A | G | C | T | C | C | T | A | A | T | T | A | A | G | T | A | A | G | A | G | C | G | T | T | C | C | C | G | T | G | C | G | A | C | C | G | A | C | C | A | G | A | A | T | C | C | T | G |
| *1 | Normal | 30 | 7.5% | A | A | A | G | C | T | T | C | C | A | A | T | T | A | A | G | T | A | A | G | A | G | C | G | T | T | C | C | C | G | T | G | C | G | A | C | C | G | A | C | C | A | G | C | A | T | C | C | T | G |
| *1 | Normal | 21 | 5.3% | A | A | A | G | C | T | C | C | T | A | A | T | G | A | G | G | T | A | A | G | A | G | C | G | T | T | C | C | C | G | T | G | C | G | A | C | C | G | A | C | C | A | G | A | A | T | C | C | T | G |
| *1 | Normal | 15 | 3.8% | A | A | A | G | C | T | T | C | C | A | A | T | T | A | A | G | T | A | A | G | A | G | C | G | T | T | C | C | C | G | T | G | C | G | A | C | C | G | A | C | C | A | G | A | A | T | C | C | T | G |
| *1 | Normal | 12 | 3.0% | A | A | A | G | C | T | C | C | T | A | A | T | T | A | A | G | T | A | A | G | G | G | C | A | T | T | C | C | C | A | T | G | T | G | G | C | C | G | C | A | C | A | A | A | A | T | C | C | T | G |
| *1 | Normal | 10 | 2.5% | A | A | A | G | C | T | C | C | T | A | A | T | T | A | A | G | T | A | A | G | A | G | C | G | T | T | C | C | C | G | T | G | C | G | A | C | C | G | A | C | C | A | G | A | A | T | C | T | C | A |
| *1 | Normal | 9 | 2.3% | A | A | A | G | C | T | C | C | T | A | A | T | T | A | A | A | T | A | A | G | A | G | C | G | T | T | C | C | C | G | T | G | C | G | A | C | C | G | A | C | C | A | G | A | A | T | C | C | T | G |
| *1 | Normal | 5 | 1.3% | A | A | A | G | C | T | C | C | T | A | A | T | T | A | A | G | T | A | A | G | A | G | C | G | T | T | C | C | C | G | T | G | C | G | A | C | C | G | A | C | C | A | G | A | T | T | C | T | T | A |
| *1 | Normal | 5 | 1.3% | A | A | A | G | C | T | C | C | T | A | A | T | T | A | A | G | T | A | A | G | A | G | C | G | T | T | T | C | C | G | C | G | C | G | A | C | C | G | A | C | C | A | G | A | A | T | C | C | T | G |
| *1 | Normal | 3 | 0.8% | A | A | A | G | C | T | C | C | T | A | A | T | G | A | G | G | T | A | A | G | G | G | C | G | C | T | C | C | C | A | T | G | T | A | A | C | C | G | A | A | T | G | G | A | T | T | C | T | T | A |
| *1 | Normal | 3 | 0.8% | A | A | A | G | C | T | C | C | T | A | A | T | T | A | A | G | T | A | A | G | A | G | C | G | T | T | C | C | C | G | T | G | C | G | A | C | C | A | A | C | C | A | G | A | A | T | C | C | T | A |
| *1 | Normal | 3 | 0.8% | A | A | A | G | C | T | C | C | T | A | A | T | T | A | A | G | T | A | A | G | G | G | C | A | T | T | C | C | C | A | T | G | T | G | G | C | C | G | C | A | C | A | A | A | A | T | C | T | T | A |
| *1 | Normal | 3 | 0.8% | A | C | A | G | C | T | C | C | T | A | A | T | T | A | A | G | T | A | A | G | A | G | C | G | T | T | C | C | C | G | T | G | C | G | A | C | C | A | A | C | C | A | G | A | A | T | C | C | T | G |
| *5 | No function | 8 | 2.0% | A | A | A | G | C | C | T | T | C | A | A | T | G | A | G | G | T | A | A | G | G | G | C | A | T | T | C | C | C | A | T | G | T | G | G | C | C | G | C | A | C | A | A | A | A | T | C | C | T | G |
| *14 | Increased | 23 | 5.8% | A | A | G | A | A | T | C | T | C | A | A | T | G | C | G | G | T | A | A | G | A | G | T | G | C | A | C | C | T | A | T | T | C | G | A | A | C | G | A | C | C | A | G | A | A | C | C | C | T | G |
| *14 | Increased | 6 | 1.5% | A | A | G | A | A | T | C | T | C | A | A | T | G | C | G | G | T | A | A | G | A | G | C | G | T | T | C | C | C | G | T | G | C | G | A | C | C | G | A | C | C | A | G | A | A | T | C | C | T | G |
| *14 | Increased | 4 | 1.0% | A | A | G | A | A | T | C | T | C | A | A | T | G | C | G | G | T | A | A | G | A | G | T | G | C | A | C | C | T | A | T | T | C | G | A | A | C | G | A | C | C | A | G | A | A | C | G | C | T | G |
| *15 | No function | 33 | 8.3% | A | A | G | G | C | C | T | T | C | A | A | T | G | A | G | G | T | A | A | G | A | G | C | G | T | T | C | C | C | G | T | G | C | G | A | C | C | G | A | C | C | A | G | A | A | T | C | C | T | G |
| *15 | No function | 21 | 5.3% | C | A | G | G | C | C | T | T | C | A | A | T | G | A | G | G | T | A | A | G | A | G | C | G | T | T | C | C | C | G | T | G | C | G | A | C | C | G | A | C | C | A | G | A | A | T | C | C | T | G |
| *15 | No function | 11 | 2.8% | C | A | G | G | C | C | T | T | C | A | A | T | G | A | G | G | T | A | A | G | G | G | C | A | T | T | C | C | C | A | T | G | T | G | G | C | C | G | C | A | C | A | A | A | A | T | C | C | T | G |
| *15 | No function | 8 | 2.0% | A | A | G | G | C | C | T | T | C | A | A | T | G | A | G | G | T | A | A | G | A | G | C | G | T | T | C | C | C | G | T | G | C | G | A | C | T | G | A | C | C | A | G | A | A | T | C | C | T | G |
| *15 | No function | 3 | 0.8% | A | A | G | G | C | C | T | T | C | A | A | T | G | A | G | G | T | A | A | G | G | G | C | A | T | T | C | C | C | A | T | G | T | G | G | C | C | G | C | A | C | A | A | A | A | T | C | C | T | G |
| *20 | Increased | 15 | 3.8% | A | A | G | G | C | T | T | T | C | G | C | T | T | A | A | G | T | A | A | G | G | G | C | A | T | T | C | C | C | A | T | G | T | G | G | C | C | G | C | A | C | A | A | A | A | T | C | T | C | A |
| *37 | Normal | 18 | 4.5% | A | A | G | G | C | T | T | T | C | A | A | C | G | A | G | G | C | C | G | C | G | A | C | G | C | T | C | C | C | A | T | G | T | A | A | A | C | G | A | A | C | G | G | A | A | T | C | T | C | A |
| *37 | Normal | 7 | 1.8% | A | A | G | G | C | T | T | T | C | A | A | T | G | C | G | G | T | A | A | G | A | G | T | G | C | A | C | T | T | A | T | T | C | G | A | A | C | G | A | C | C | A | G | A | A | C | C | C | T | G |
| *37 | Normal | 6 | 1.5% | A | A | G | G | C | T | T | T | C | A | A | C | G | A | G | G | C | C | G | C | G | A | C | G | C | T | C | C | C | A | T | G | T | A | A | A | C | G | A | A | C | G | G | A | A | T | C | C | T | G |

**Figure S7** *SLCO1B1-SLCO1A2* haplotypes in 200 healthy unrelated Finnish participants from a previous pharmacogenetic study.[27] Haplotypes with less than three observations were excluded from the figure. Missense variants are marked with dark green color and other variants with light green color. CPIC, Clinical Pharmacogenetics Implementation Consortium

**References**


1. Kajosaari, L. I., Backman, J. T., Neuvonen, M., Laitila, J. & Neuvonen, P. J. Lack of effect of bezafibrate and fenofibrate on the pharmacokinetics and pharmacodynamics of repaglinide. *Br. J. Clin. Pharmacol.* **58**, 390–396 (2004).
2. Kajosaari, L. I. *et al.* Cyclosporine markedly raises the plasma concentrations of repaglinide. *Clin. Pharmacol. Ther.* **78**, 388–399 (2005).
3. Kalliokoski, A., Neuvonen, M., Neuvonen, P. J. & Niemi, M. Different effects of SLCO1B1 polymorphism on the pharmacokinetics and pharmacodynamics of repaglinide and nateglinide. *J. Clin. Pharmacol.* **48**, 311–321 (2008).
4. Kalliokoski, A., Backman, J. T., Neuvonen, P. J. & Niemi, M. Effects of the SLCO1B1*1B haplotype on the pharmacokinetics and pharmacodynamics of repaglinide and nateglinide. *Pharmacogenet. Genomics* **18**, 937–942 (2008).
5. Kalliokoski, A., Neuvonen, M., Neuvonen, P. J. & Niemi, M. The effect of SLCO1B1 polymorphism on repaglinide pharmacokinetics persists over a wide dose range. *Br. J. Clin. Pharmacol.* **66**, 818–825 (2008).
6. Tornio, A. *et al.* Glucuronidation converts clopidogrel to a strong time-dependent inhibitor of CYP2C8: a phase II metabolite as a perpetrator of drug-drug interactions. *Clin. Pharmacol. Ther.* **96**, 498–507 (2014).
7. Kajosaari, L. I., Jaakkola, T., Neuvonen, P. J. & Backman, J. T. Pioglitazone, an in vitro inhibitor of CYP2C8 and CYP3A4, does not increase the plasma concentrations of the CYP2C8 and CYP3A4 substrate repaglinide. *Eur. J. Clin. Pharmacol.* **62**, 217–223 (2006).
8. Kajosaari, L. I., Niemi, M., Backman, J. T. & Neuvonen, P. J. Telithromycin, but not montelukast, increases the plasma concentrations and effects of the cytochrome P450 3A4 and 2C8 substrate repaglinide. *Clin. Pharmacol. Ther.* **79**, 231–242 (2006).
9. Niemi, M., Kajosaari, L. I., Neuvonen, M., Backman, J. T. & Neuvonen, P. J. The CYP2C8 inhibitor trimethoprim increases the plasma concentrations of repaglinide in healthy subjects. *Br. J. Clin. Pharmacol.* **57**, 441–447 (2004).
10. Niemi, M., Backman, J. T., Neuvonen, M. & Neuvonen, P. J. Effects of gemfibrozil, itraconazole, and their combination on the pharmacokinetics and pharmacodynamics of repaglinide: potentially hazardous interaction between gemfibrozil and repaglinide. *Diabetologia* **46**, 347–351 (2003).

11. Kalliokoski, A., Backman, J. T., Kurkinen, K. J., Neuvonen, P. J. & Niemi, M. Effects of gemfibrozil and atorvastatin on the pharmacokinetics of repaglinide in relation to SLCO1B1 polymorphism. *Clin. Pharmacol. Ther.* **84**, 488–496 (2008).
12. Tornio, A. *et al.* The effect of gemfibrozil on repaglinide pharmacokinetics persists for at least 12 h after the dose: evidence for mechanism-based inhibition of CYP2C8 in vivo. *Clin. Pharmacol. Ther.* **84**, 403–411 (2008).
13. Backman, J. T. *et al.* CYP2C8 activity recovers within 96 hours after gemfibrozil dosing: estimation of CYP2C8 half-life using repaglinide as an in vivo probe. *Drug Metab. Dispos.* **37**, 2359–2366 (2009).
14. Honkalammi, J., Niemi, M., Neuvonen, P. J. & Backman, J. T. Mechanism-based inactivation of CYP2C8 by gemfibrozil occurs rapidly in humans. *Clin. Pharmacol. Ther.* **89**, 579–586 (2011).
15. Honkalammi, J., Niemi, M., Neuvonen, P. J. & Backman, J. T. Dose-dependent interaction between gemfibrozil and repaglinide in humans: strong inhibition of CYP2C8 with subtherapeutic gemfibrozil doses. *Drug Metab. Dispos.* **39**, 1977–1986 (2011).
16. Honkalammi, J., Niemi, M., Neuvonen, P. J. & Backman, J. T. Gemfibrozil is a strong inactivator of CYP2C8 in very small multiple doses. *Clin. Pharmacol. Ther.* **91**, 846–855 (2012).
17. Karonen, T., Neuvonen, P. J. & Backman, J. T. CYP2C8 but not CYP3A4 is important in the pharmacokinetics of montelukast. *Br. J. Clin. Pharmacol.* **73**, 257–267 (2012).
18. Karonen, T. *et al.* Gemfibrozil markedly increases the plasma concentrations of montelukast: a previously unrecognized role for CYP2C8 in the metabolism of montelukast. *Clin. Pharmacol. Ther.* **88**, 223–230 (2010).
19. Karonen, T., Neuvonen, P. J. & Backman, J. T. The CYP2C8 inhibitor gemfibrozil does not affect the pharmacokinetics of zafirlukast. *Eur. J. Clin. Pharmacol.* **67**, 151–155 (2011).
20. Filppula, A. M., Tornio, A., Niemi, M., Neuvonen, P. J. & Backman, J. T. Gemfibrozil impairs imatinib absorption and inhibits the CYP2C8-mediated formation of its main metabolite. *Clin. Pharmacol. Ther.* **94**, 383–393 (2013).
21. Backman, J. T., Luurila, H., Neuvonen, M. & Neuvonen, P. J. Rifampin markedly decreases and gemfibrozil increases the plasma concentrations of atorvastatin and its metabolites. *Clin. Pharmacol. Ther.* **78**, 154–167 (2005).

22. Tornio, A., Niemi, M., Neuvonen, P. J. & Backman, J. T. Stereoselective interaction between the CYP2C8 inhibitor gemfibrozil and racemic ibuprofen. *Eur. J. Clin. Pharmacol.* **63**, 463–469 (2007).
23. Niemi, M. *et al.* Itraconazole, gemfibrozil and their combination markedly raise the plasma concentrations of loperamide. *Eur. J. Clin. Pharmacol.* **62**, 463–472 (2006).
24. Niemi, M. *et al.* Gemfibrozil considerably increases the plasma concentrations of rosiglitazone. *Diabetologia* **46**, 1319–1323 (2003).
25. Tornio, A., Neuvonen, P. J. & Backman, J. T. The CYP2C8 inhibitor gemfibrozil does not increase the plasma concentrations of zopiclone. *Eur. J. Clin. Pharmacol.* **62**, 645–651 (2006).
26. Auton, A. *et al.* A global reference for human genetic variation. *Nature* **526**, 68–74 (2015).
27. Hirvensalo, P. *et al.* Enantiospecific pharmacogenomics of fluvastatin. *Clin. Pharmacol. Ther.* **106**, 668–680 (2019).
